# Centralized scheduling, decentralized scheduling or demand scheduling? How to more effectively allocate and recycle shared takeout lunch boxes

**DOI:** 10.1371/journal.pone.0319257

**Published:** 2025-03-04

**Authors:** Yuntao Bai, Di Liu, Jili Ma

**Affiliations:** 1 Business School, Shandong Management University, Jinan, China; 2 Center for Industrial and Business Organization, Dongbei University of Finance and Economics, Dalian, China; 3 School of Transportation and Logistics Engineering, Shandong Jiaotong University, Jinan, China; Alma Mater Studiorum Universita di Bologna: Universita degli Studi di Bologna, ITALY

## Abstract

Efficient scheduling of shared takeaway containers plays a significant role in the sharing economy system. An effective scheduling system ensures the maximization of container reuse, reducing resource waste and environmental pollution. To explore the applicability of different scheduling models for shared takeaway containers, this paper constructs differential game models for three modes: centralized scheduling, decentralized scheduling, and demand-based scheduling. The equilibrium outcomes are compared and analyzed. The research findings indicate that when the revenue from scheduling takeaway containers is low, decentralized scheduling can yield the maximum benefit for takeaway platforms; conversely, when the revenue is high, centralized scheduling offers the greatest benefit. For restaurant enterprises, when the revenue from scheduling is low, if the cost of scheduling is also low, demand-based scheduling can provide the maximum benefit; however, if the cost is high, decentralized scheduling is more advantageous; otherwise, centralized scheduling can maximize the benefits for restaurant enterprises.

## 1. Introduction

The efficient scheduling of shared meal boxes in the sharing economy is of paramount importance. An effective scheduling system ensures the maximization of meal box reuse, reducing resource waste and environmental pollution. Through intelligent scheduling, meal boxes can circulate efficiently across different regions, preventing resource idleness and waste due to improper scheduling. Efficient scheduling reduces logistics and delivery costs, increases the turnover rate of meal boxes, and thereby lowers operational expenses. The effective use of meal boxes enhances the service efficiency of catering businesses, increases order processing capacity, and ultimately improves profitability [1]. An efficient scheduling system ensures that users receive meal boxes promptly, enhancing user experience and satisfaction. Through intelligent scheduling, users can access and return shared meal boxes at any time and place, enjoying more convenient services. Advanced technological support, such as big data analytics, the Internet of Things, and artificial intelligence, is essential for efficient scheduling, which will promote continuous innovation and development in related technologies. An efficient scheduling system, through real-time analysis and management of large amounts of data, provides strong support for operational decision-making and improves management efficiency [2]. The shared meal box usage model, driven by efficient scheduling, can encourage consumers to develop good environmental habits and raise environmental awareness across society. The concept of the sharing economy is fully embodied through efficient meal box scheduling, contributing to the fair distribution of resources and promoting social harmony. An efficient scheduling system better aligns with government environmental policy requirements, facilitating policy implementation. Through efficient scheduling, the standardized management of shared meal boxes can be achieved, ensuring hygiene and safety during use. In conclusion, the efficient scheduling of shared meal boxes not only optimizes resource utilization and reduces operational costs but also enhances user experience, promotes technological advancement, and fosters social harmony. Efficient scheduling is key to the sustainable development of shared meal boxes and is an important means of advancing an environmentally friendly economy.

With the continued deepening of research into scheduling issues, a significant body of work has emerged. Some scholars have focused on medical scheduling and health management. For instance, Kianfar and Atighehchian [3] applied a hybrid heuristic approach to surgical scheduling; Bazirha et al. [4] analyzed the stochastic home healthcare routing and scheduling problem involving multiple synchronized services; Zhu et al. [5] considered the transportation and deterioration scenarios in mass casualty incidents to schedule operating rooms across multiple hospitals; Koruca et al. [6] developed a novel personalized employee scheduling method from a work-life balance perspective, using hospitals as a case study; and Sauré et al. [7] analyzed the dynamic multi-priority, multi-class patient scheduling problem with stochastic service times. These studies examine medical scheduling and health management from various perspectives, including surgical scheduling, healthcare scheduling, hospital staff scheduling, and patient scheduling.

Some scholars have focused on the scheduling issues within power and energy systems. For example, Shi et al. [8] explored the carbon-oriented optimal scheduling of electric vehicle aggregators using a Lagrangian-enhanced safe reinforcement learning algorithm; Kapoor et al. [9] achieved optimal scheduling of electric vehicles through centralized and decentralized pricing strategies; Dong et al. [10] conducted wind-hydro-thermal joint scheduling based on the symbiotic organism search algorithm; Santos et al. [11] applied accelerated dual dynamic integer programming to analyze short-term generation scheduling; Chowdhury [12] studied a coordinated electric vehicle (EV) charging scheduling scheme based on charging time and resource cost awareness; Li et al. [13] optimized the scheduling of isolated microgrids through multi-period forecasting based on automated reinforcement learning; and Hjelmeland et al. [14] investigated non-convex mid-term hydropower scheduling based on stochastic dual dynamic integer programming. These studies primarily examine power and energy system scheduling issues from various aspects, including carbon-oriented optimal scheduling, electric vehicle scheduling, wind-hydro-thermal joint scheduling, short-term generation scheduling, charging scheduling, microgrid scheduling, and hydropower scheduling.

Some scholars have focused on scheduling issues in production and industrial processes. For instance, Lu et al. [15] studied the scheduling of manufacturing cells in permutation flow shops; Missaoui and Ruiz [16] addressed hybrid flow shop scheduling problems with setup times and deadline windows using a parameterless iterative greedy method; Milenković et al. [17] explored simultaneous batch sizing and scheduling in the animal feed premix industry; Al-E-Hashem et al. [18] applied bi-objective optimization to develop robust maintenance planning and scheduling for multi-factory production networks; Smutnicki et al. [19] analyzed cyclic flow shop scheduling problems with no-wait constraints and missing operations; and Kurowski et al. [20] used a quantum approximate optimization algorithm to examine job shop scheduling problems. These studies primarily investigate production and industrial scheduling issues from various perspectives, including manufacturing cell scheduling, hybrid flow shop scheduling, animal feed scheduling, production network scheduling, cyclic flow shop scheduling, and job shop scheduling.

Some scholars have focused on logistics and transportation scheduling. For instance, Frisch et al. [21] analyzed the integrated truck routing and train scheduling problem; Xu et al. [22] developed a simulation-based multi-objective model for flexible job shop transportation scheduling; Albornoz et al. [23] studied the planning and scheduling of selective logging through the delineation of management zones; and Lakzaei et al. [24] used a novel hybrid solution to optimize the integrated scheduling and routing of post-disaster repair crews and rescue vehicles. These studies primarily investigate logistics and transportation scheduling issues from various perspectives, including truck routing, flexible job shop transportation, selective logging, and rescue vehicle operations.

Some scholars have focused on scheduling issues in computing and information systems. For example, Li [25] studied task bundle scheduling in large computing systems; Lee and Lee [26] addressed sequence sizing and scheduling problems using a novel integer optimization model and approximate dynamic programming algorithm; Polten and Emde [27] analyzed multi-shuttle crane scheduling in automated storage and retrieval systems; Esmaelian et al. [28] examined the problem of scheduling capacitive identical parallel machines; and Yim et al. [29] explored reducing inversion intervals in single-machine scheduling. These studies primarily investigate scheduling issues in computing and information systems from various perspectives, including task bundles, sequence sizing, multi-shuttle cranes, and parallel machines.

Despite significant achievements in the fields of healthcare scheduling, energy system scheduling, production and industrial scheduling, logistics and transportation scheduling, as well as computing and information systems scheduling, there remain some critical research gaps in the area of shared takeaway container scheduling. The first gap lies in the insufficient integration of cross-disciplinary scheduling methods. Most existing research focuses on optimization within specific domains, lacking the integration and application of advanced scheduling methods from different fields to new contexts. For example, the application of optimization algorithms and techniques from areas such as healthcare scheduling, energy system scheduling, and logistics scheduling to the scheduling of shared takeaway containers has not yet been fully explored.

The second gap is the inadequate design of scheduling systems within the sharing economy model. Although the sharing economy has been applied in various industries, there is limited research on the design of scheduling systems for shared takeaway containers. In particular, how to design an efficient scheduling system within a multi-stakeholder sharing economy model that coordinates the demands and resource allocation among different stakeholders has not been thoroughly explored.

The third gap is the lack of research on the trade-offs between centralized and decentralized scheduling. Existing studies have primarily focused on the advantages and disadvantages of each approach individually, but there is insufficient research on balancing and optimizing these two scheduling models in the context of shared takeaway container scheduling. In particular, systematic studies on how to dynamically adjust the ratio of centralized to decentralized scheduling based on actual needs are still lacking.

In light of these gaps, the main contributions and innovations of this paper are as follows. First, the innovative application of cross-disciplinary scheduling methods. By integrating and applying advanced scheduling algorithms and techniques from fields such as healthcare, power systems, and logistics to the scheduling of shared takeaway containers, this research pioneers a new cross-disciplinary scheduling application model. This model will help address complex scheduling challenges within the sharing economy, enhancing resource utilization efficiency and service levels.

Second, the design of an intelligent scheduling system within the sharing economy model. By designing an intelligent scheduling system that aligns with the characteristics of the sharing economy, the system can effectively coordinate the demands and resource allocation among delivery platforms, food service businesses, and users. This will provide a new design framework for scheduling systems in the sharing economy, enhancing overall operational efficiency.

Third, the dynamic trade-off and optimization between centralized and decentralized scheduling. By conducting an in-depth analysis of the advantages and disadvantages of both centralized and decentralized scheduling, this study proposes a method for dynamically adjusting scheduling strategies, enabling delivery platforms and food service businesses to adapt their scheduling models based on real-time demand. This contribution will significantly enhance the flexibility and adaptability of scheduling systems.

This study is of significant importance. The role of an efficient scheduling system in the management of shared takeaway containers cannot be underestimated. By optimizing and innovating centralized scheduling, decentralized scheduling, and demand-based scheduling, it is possible to address issues such as resource waste, high costs, and poor user experience, thereby promoting comprehensive improvements in the management of shared takeaway containers and achieving a win-win situation in terms of economic and social benefits. This research is both theoretically and practically significant, offering new insights and methods for resource scheduling within the sharing economy model. Effectively scheduling shared takeaway containers not only optimizes resource utilization, reduces operational costs for businesses, and enhances user experience, but also promotes technological innovation and social harmony. It is crucial for the sustainable development of shared takeaway containers and serves as an important means of advancing an environmentally-friendly economy.

## 2. Methodology

### 2.1 Problem description, hypothesis, and variable definition

#### 2.1.1 Problem description.

In the process of efficiently scheduling shared takeaway boxes, selecting takeaway platforms and catering enterprises as the primary stakeholders is mainly due to their crucial roles in the entire takeaway ecosystem and the mutual influence of their interests and decisions. The takeaway platform acts as a bridge between consumers and catering enterprises, responsible for key aspects such as order management, delivery scheduling, and user experience. With access to vast amounts of data and resources, the platform can effectively drive the implementation and optimization of shared takeaway boxes. Catering enterprises are the direct users and providers of takeaway boxes, and their operating costs and service efficiency are directly affected by the scheduling efficiency of the boxes. The participation and cooperation of catering enterprises are necessary conditions for the smooth operation of shared takeaway boxes. Both takeaway platforms and catering enterprises aim to reduce costs, improve efficiency, and enhance user experience, thus sharing common interests in the use of shared takeaway boxes.

However, there may be disagreements on specific operations and cost-sharing, such as the costs of purchasing and maintaining shared boxes and the management and coordination of deliveries, which require clear delineation of responsibilities and benefits. Takeaway platforms, with their access to large amounts of user data, order data, and logistics information, can achieve efficient scheduling and optimization of the boxes through big data analysis. Catering enterprises, with numerous terminal outlets and daily operational practices, can provide practical usage data and feedback to help optimize scheduling strategies. Effective scheduling necessitates close collaboration between takeaway platforms and catering enterprises, with both parties jointly developing and executing strategies for the use and recovery of shared boxes to maximize resource utilization. Through mutual negotiation and cooperation, a more rational management system and operational processes can be established, ensuring the efficient and smooth use of shared boxes [24].

When the government promotes environmental policies, takeaway platforms and catering enterprises, as the main executors, need to jointly respond to policy requirements and promote the widespread use of shared takeaway boxes. As industry leaders, takeaway platforms and catering enterprises should assume a certain degree of social responsibility, promoting green development and sustainable operations to create a positive social impact. In summary, selecting takeaway platforms and catering enterprises as the primary stakeholders can fully leverage their advantages, achieve efficient scheduling and sustainable development of shared takeaway boxes through collaborative cooperation and reasonable distribution of benefits.

The game between takeaway platforms and catering enterprises is continuous, sustained, and dynamic for several reasons. Firstly, changes in the market environment. The demand in the takeaway market fluctuates with factors such as seasons, holidays, and weather, requiring adjustments in the scheduling of shared boxes, making the game continuous and dynamic; the intense market competition compels takeaway platforms and catering enterprises to continually optimize their services and operational strategies to respond to competitors’ challenges, resulting in an evolving game. Secondly, technological advancements and data updates. The continuous advancement of technologies such as big data, the Internet of Things, and artificial intelligence provides new tools and methods for the scheduling of shared boxes, necessitating adjustments and optimizations in the game process in line with technological progress; user behavior and demand data are dynamically changing, requiring takeaway platforms and catering enterprises to constantly analyze and utilize the latest data to optimize scheduling strategies [30]. Thirdly, interest adjustment and coordination. The scheduling of shared boxes involves multiple interests, and takeaway platforms and catering enterprises need to continually negotiate and adjust cost-sharing and benefit distribution to achieve a dynamic balance; changes in government policies and regulations also affect the scheduling strategies of shared boxes, requiring both parties to adjust according to the latest policies to maintain a dynamic game. Fourthly, user experience and feedback. User needs and preferences are continuously changing, necessitating takeaway platforms and catering enterprises to constantly optimize services based on user feedback and adjust the scheduling strategies of shared boxes; to enhance user experience, both parties need to continually improve service processes and quality, which is a continuous and dynamic game. Fifthly, external environment and emergencies. Emergencies such as pandemics and natural disasters affect the operation of the takeaway business, requiring takeaway platforms and catering enterprises to dynamically adjust scheduling strategies to cope with sudden situations [24]; changes in macroeconomic and policy environments affect the use and scheduling of shared boxes, necessitating continuous dynamic adjustments by both parties. In summary, in the process of scheduling shared takeaway boxes, takeaway platforms and catering enterprises must engage in continuous, sustained, and dynamic games to address challenges and impacts from changes in the market environment, technological advancements, and user demand, ensuring the effectiveness and sustainability of scheduling strategies.

In the process of effectively scheduling shared takeaway boxes, takeaway platforms and catering enterprises primarily use three models: centralized scheduling, decentralized scheduling, and demand-driven scheduling.

(1) Centralized Scheduling. The centralized scheduling model refers to the takeaway platform managing and scheduling the use, distribution, recovery, and cleaning of shared takeaway boxes through a centralized system or center [16]. This model aims to optimize resource allocation, improve operational efficiency, reduce costs, and ensure the efficient cyclic use of shared boxes. The centralized scheduling model has several characteristics. Firstly, centralized management. Through a centralized management system, the takeaway platform can uniformly coordinate the shared box demands and supplies of different catering enterprises, avoiding resource waste. The centralized management system can monitor the usage of shared boxes in real-time, including the number of boxes, their usage status, and recovery progress, ensuring timely and accurate information. Secondly, optimized resource allocation. Utilizing big data and predictive algorithms, the centralized scheduling system can forecast the demand for boxes at different times and locations, allowing for preemptive preparation. Through centralized management, the takeaway platform can effectively manage the inventory of shared boxes, ensuring adequate supply during peak periods while avoiding excessive inventory buildup. Thirdly, efficient recovery and cleaning. The centralized scheduling system can optimize the routes and timing of box recovery based on real-time data, enhancing recovery efficiency. Recovered boxes are concentrated at specific locations for cleaning and disinfection, leveraging economies of scale to reduce cleaning costs and ensure quality. By means of unified coordination, optimized resource allocation, and efficient recovery and cleaning, the centralized scheduling model improves the operational efficiency and service quality of shared takeaway boxes while reducing operational costs [26]. However, challenges such as technological infrastructure, data security, and inter-enterprise cooperation need to be overcome in implementation. Through continuous optimization and improvement, the centralized scheduling model can bring significant economic and environmental benefits to takeaway platforms and catering enterprises.
(2) Decentralized Scheduling. The decentralized scheduling model refers to takeaway platforms and catering enterprises independently managing and scheduling the use, distribution, recovery, and cleaning of shared takeaway boxes within their own management systems [17]. This model emphasizes independent operation to flexibly respond to market changes and personalized demands. The main characteristics of the decentralized scheduling model are as follows. First, independent management. Each catering enterprise or takeaway platform independently schedules and manages the use and recovery of shared boxes based on its own needs and operational conditions. Enterprises can localize optimizations and flexibly adjust scheduling strategies according to specific situations. Second, flexible response. Enterprises can quickly respond to changes in market demand and adjust box distribution and recovery plans promptly. They can provide personalized box usage and recovery services according to the needs of different customer groups. Third, diversified cooperation. Multiple catering enterprises and takeaway platforms are encouraged to participate in promoting the use and management of shared boxes. Enterprises can collaborate flexibly based on actual circumstances, forming diversified cooperation models. By means of independent management, flexible response, and diversified cooperation, the decentralized scheduling model enhances the flexibility and market adaptability of shared takeaway boxes while reducing the systemic risks associated with centralized management [9]. However, challenges such as resource coordination, data sharing and security, and operational efficiency need to be addressed in the implementation process. Through effective management and coordination, the decentralized scheduling model can provide takeaway platforms and catering enterprises with a flexible and efficient operational approach, promoting the widespread adoption and application of shared boxes.
(3) Demand-Driven Scheduling. The demand-driven scheduling model dynamically adjusts the allocation, usage, recovery, and cleaning of shared takeaway boxes based on real-time data on orders and box demand. This model emphasizes a demand-oriented approach, utilizing data analysis and intelligent scheduling systems to achieve optimal resource allocation and efficient management [16]. The demand-driven scheduling model has the following characteristics. First, real-time data-driven. Relevant data on takeaway orders and box usage are collected in real-time through order systems, user feedback, sensors, and other channels. Utilizing big data and artificial intelligence technologies, the collected data is analyzed in real-time to predict and identify changes in demand. Second, intelligent scheduling system. Based on the data analysis results, the intelligent scheduling system can dynamically adjust the allocation and recovery plans of boxes, optimizing resource allocation. Through automation technology, the efficient management of the allocation, usage, recovery, and cleaning of boxes is achieved. Third, flexible response to demand. According to real-time demand, the quantity and location of box allocation can be flexibly adjusted to ensure adequate supply. In cases of significant demand fluctuations, the system can quickly respond and timely adjust the scheduling plan to avoid resource waste and supply shortages. By means of real-time data-driven, intelligent scheduling systems, and flexible response to demand, the demand-driven scheduling model improves the resource utilization rate, service quality, and operational efficiency of shared takeaway boxes. However, challenges such as data accuracy, technological investment, and cross-departmental coordination need to be addressed in the implementation process [4]. Through effective management and continuous optimization, the demand-driven scheduling model can bring significant economic and environmental benefits to takeaway platforms and catering enterprises, promoting the widespread adoption and application of shared boxes.

**Fig 1:**
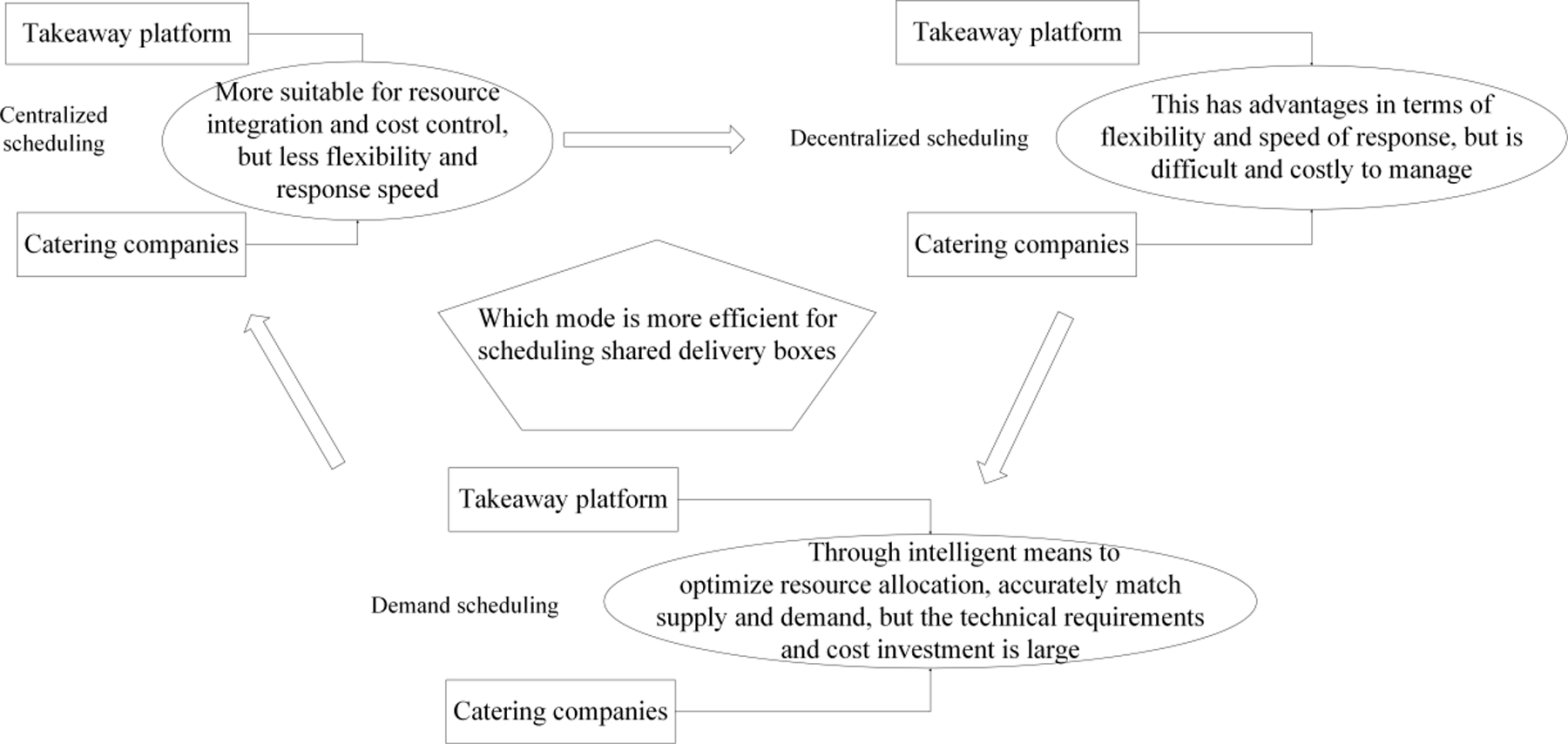
Relationship between three dispatch mode of takeaway lunch boxes.

The relationship between the three dispatch mode of takeaway lunch boxes is shown in Fig 1.

#### 2.1.2 Hypothesis.

Hypothesis 1: Shared takeaway boxes undergo sterilization and disinfection, and there are no contamination issues during the scheduling process.

In the process of effectively scheduling shared takeaway boxes, ensuring that these boxes are thoroughly sterilized and disinfected, and that there are no contamination issues during the scheduling process, is a crucial step. To achieve this, the following measures are necessary. First, strict sterilization and disinfection procedures. Shared takeaway boxes must undergo a standardized sterilization and disinfection process after each use. Various effective methods such as high-temperature disinfection, ultraviolet disinfection, ozone disinfection, or food-grade disinfectants should be employed to ensure thorough disinfection. Establishing dedicated cleaning and disinfection centers for the centralized cleaning and disinfection of shared boxes can ensure that each box undergoes a standardized process, eliminating potential health risks. Second, fully enclosed transportation and storage. The disinfected shared takeaway boxes should be sealed with hygienic packaging materials to prevent secondary contamination during transportation and storage. During storage and transportation, aseptic environments or specialized sterile containers should be used to ensure that the boxes remain sterile before use, avoiding external contamination. Third, intelligent monitoring and traceability system. An intelligent monitoring system should be used to monitor in real-time the cleaning, disinfection, packaging, storage, and transportation of the boxes, ensuring that each step meets hygiene standards. A comprehensive traceability system should be established to record detailed cleaning and disinfection logs for each box, allowing users and management to access this information at any time, ensuring transparency and trustworthiness. Through these measures, it can be ensured that shared takeaway boxes, after undergoing strict sterilization and disinfection, will not be contaminated during the scheduling process.

Hypothesis 2: Shared takeaway food containers have a certain usage proportion in the local area.

In the process of effectively scheduling shared takeaway food containers, having a certain usage proportion of these containers locally has multiple positive impacts on the system’s operation and management. First, economies of scale. When the usage proportion of shared takeaway food containers reaches a certain level locally, economies of scale can be generated, enhancing the overall system’s utilization and efficiency. A higher usage proportion means more user participation, which helps optimize the scheduling and recycling of containers. Economies of scale also help reduce the cost of cleaning and distributing the containers per use, making the operation of shared takeaway food containers more economical. Second, a stable user base. A certain usage proportion reflects the market’s recognition and demand for shared takeaway food containers. This stable user base can support the continuous operation and development of shared takeaway food containers. A higher usage proportion helps form user habits, increasing users’ reliance and stickiness to shared containers, further enhancing the usage rate. Third, improved supporting facilities. With a certain usage proportion, it is more reasonable to set up recycling points and cleaning centers for shared takeaway food containers locally, facilitating the return and recycling of the containers. A high usage proportion encourages takeaway platforms and delivery companies to optimize logistics networks, improve delivery efficiency, and ensure timely recycling and reuse of containers. In summary, achieving a certain usage proportion of shared takeaway food containers locally plays a significant role in promoting the efficient scheduling and management of the system.

Hypothesis 3: Information technology is fully utilized in the local area.

In the process of effectively scheduling shared takeaway food containers, information technology is fully utilized in the local area, playing a critical role in enhancing scheduling efficiency, ensuring container hygiene, and optimizing user experience. First, big data and artificial intelligence technologies are used to analyze takeaway orders, user demands, and delivery routes in real time, optimizing container scheduling and delivery paths to reduce delivery time and costs. An intelligent scheduling platform is established to automatically match orders with shared containers, ensuring efficient use and timely delivery of containers. Second, GPS devices are installed on delivery vehicles and shared containers for real-time tracking, ensuring a transparent and controllable delivery process to prevent loss and delays. Monitoring cameras and sensors are utilized to provide real-time surveillance of cleaning and disinfection centers, and storage centers, ensuring that every stage meets hygiene standards. Third, user-end applications. Through mobile applications, users can conveniently order food, choose to use shared containers, and track their order status in real-time to understand the delivery progress of the containers. Users can submit feedback and reviews through the application, helping the platform understand user needs and issues promptly for improvement and optimization. By fully utilizing information technology, the scheduling process of shared takeaway food containers has been greatly optimized and enhanced. The comprehensive application of these information technologies not only improves scheduling efficiency and accuracy but also ensures the hygiene and safety of the containers, optimizes the user experience, and promotes the successful implementation and dissemination of shared takeaway food containers in the local area.

#### 2.1.3 Variable definition.

When constructing the differential game model in this article, many parameters and variables are designed. These parameters and variables are defined as shown in Table 1.

**Table 1:** The main definition of variables and parameters in this article.

Variables and parameters	Specific meaning
*Y = *{*C,D,B*}	Dispatch mode of takeaway lunch boxes (centralized scheduling, decentralized scheduling or demand scheduling)
Independent variable	
*F*_*Y*1_(*t*)	Takeaway platform’s efforts to dispatch takeaway lunch boxes under the dispatch mode *Y*
*F*_*Y*2_(*t*)	Catering companies’ efforts to dispatch takeaway lunch boxes under the dispatch mode *Y*
*x*_*Y*1_(*t*)	Reputation of takeaway platform in the dispatch process of takeaway lunch boxes under the dispatch mode *Y*
*x*_*Y*2_(*t*)	Reputation of catering companies in the dispatch process of takeaway meal boxes under the dispatch mode *Y*
Parameter	
ρ	The discount rate that occurs over time, 0≤ρ≤1
δ	Decay of reputation, δ>0
*a*_1_, *a*_2_	The benefits that the takeaway platform or catering companies gain by dispatching takeaway lunch boxes at a unit level, *a*_1_, *a*_2_ > 0
*c*_1_,*c*_2_	The cost to the takeaway platform or catering companies of dispatching takeaway lunch boxes at a unit level, *c*_1_, *c*_2_ > 0
*f*_1_, *f*_2_	The reputation that the takeaway platform or catering companies gain by dispatching takeaway lunch boxes at a unit level, *f*_1_, *f*_2_ > 0
*a*_*C*_	Gains from resource consolidation by takeaway platform, *a*_*C*_ > 0
*b*_*C*_	Impact factor of cost control on catering companies, *b*_*C*_ > 0
*f*_*C*_	Impact factor of lower responsiveness on takeout platforms or caterers, *f*_*C*_ > 0
*a*_*D*_	Reduced revenue due to increased difficulty in managing takeaway platforms, *a*_*D*_ > 0
*c*_*D*_	Increased costs to catering companies resulting from decentralized scheduling, *c*_*D*_ > 0
*f*_*D*_	Reputational impact of improvements in flexibility and responsiveness, *f*_*D*_ > 0
*a*_*B*_	Increased returns from investing in technology, *a*_*B*_ > 0
*c*_*B*_	Cost of investing in technology, *c*_*B*_ > 0
Function	
*J*_*Y*1_(*t*)	The social welfare function of takeaway platform under the dispatch mode *Y*
*J*_*Y*2_(*t*)	The social welfare function of catering companies under the dispatch mode *Y*
*V*_*Y*1_(*t*)	The social benefits of takeaway platform under the dispatch mode *Y*
*V*_*Y*2_(*t*)	The social benefits of catering companies under the under the dispatch mode *Y*

### 2.2 Differential game of three dispatch modes

In the process of efficiently coordinating shared takeaway containers between delivery platforms and food service businesses, a centralized scheduling model is one where resources are managed and allocated through a centralized system. While centralized scheduling is better suited for resource integration and cost control, it is less flexible and slower to respond. This model consolidates all scheduling and management of shared takeaway containers into a unified system or organization, enabling a high degree of resource integration. This approach avoids the resource fragmentation and redundant investments that can occur when individual food service businesses or platforms manage resources independently, thereby improving resource utilization. Through centralized scheduling, operations such as cleaning, sanitization, distribution, and retrieval of containers can be standardized, ensuring consistency in quality across each stage. This contributes to improved operational efficiency and guarantees the hygiene and safety of the containers. The centralized scheduling model can achieve lower costs through large-scale centralized procurement, cleaning, and sanitization services. Due to centralized processing, the cost per container can be significantly reduced, thereby enhancing overall economic efficiency [31]. Centralized scheduling also simplifies management processes and improves efficiency, reducing the human and financial resources required for independent management by each participant. Platforms or businesses can utilize a unified management system to achieve full-process tracking and control of the containers, further reducing management costs.

Given that centralized scheduling involves multiple stages and processes, if demand changes unexpectedly—such as during sudden order surges or special events—the centralized scheduling system may struggle to respond in a timely manner, leading to shortages of containers or scheduling delays, which can negatively impact the user experience [9]. The centralized scheduling model focuses on unified management of overall resources, making it difficult to flexibly adjust to the specific needs of individual food service businesses or users. Consequently, this may reduce the level of service personalization, making it less favorable for meeting the demands of different market segments. Centralized scheduling implies that key resources and management are concentrated within a single system or organization. Should this central entity encounter issues—such as system failures or management errors—the entire container scheduling system could be significantly impacted, potentially leading to a disruption of the entire supply chain. Furthermore, centralized scheduling requires the coordination of multiple stakeholders, including food service businesses and logistics providers, which incurs higher communication costs. If coordination is inadequate, it could lead to a decline in scheduling efficiency and even trigger conflicts of interest among the parties involved.

If takeaway platform and catering companies dispatch shared takeout boxes through the mode of centralized scheduling, then their social welfare function can be expressed as:


$$J_{C1}=\int_{0}^{\infty } [\left(a_{1}+a_{C}\right)F_{C1}\left(t\right)-\frac{c_{1}}{2}F_{C1}^{2}\left(t\right)\left.+lx_{C1}\left(t\right)\right]\;e^{-\rho t}dt$$ (1)

$$J_{C2}=\int_{0}^{\infty } [a_{2}F_{C2}\left(t\right)-\frac{c_{2}}{2\ln\left(\text{e}+b_{C}\right)}F_{C2}^{2}\left(t\right)\left.+lx_{C2}\left(t\right)\right]\;e^{-\rho t}dt$$ (2)

In the above formula, $a_{1}F_{C1}\left(t\right)$ represents the benefits obtained by the delivery platform from the centralized scheduling of shared delivery boxes. $a_{C}F_{C1}\left(t\right)$ represents the benefits gained by the delivery platform through resource integration under the centralized scheduling model. $\frac{c_{1}}{2}F_{C1}^{2}\left(t\right)$ represents the costs incurred by the delivery platform for the centralized scheduling of shared delivery boxes. $lx_{C1}\left(t\right)$ indicates the positive impact of the delivery platform’s reputation. $a_{2}F_{C2}\left(t\right)$ represents the benefits obtained by catering enterprises from the centralized scheduling of shared delivery boxes. $\frac{c_{2}}{2\ln\left(\text{e}+b_{C}\right)}F_{C2}^{2}\left(t\right)$ represents the costs incurred by catering enterprises for the centralized scheduling of shared delivery boxes. $\ln\left(\text{e}+b_{C}\right)$ indicates the impact of cost control on catering enterprises. $lx_{C2}\left(t\right)$ represents the positive impact of the reputation of catering enterprises.

The change in the reputation of takeaway platform and catering companies under the mode of centralized scheduling can be expressed as:


$${\dot{x}}_{C1}\left(t\right)=\left(f_{1}-f_{C}\right)F_{C1}\left(t\right)-\delta x_{C1}\left(t\right)$$ (3)

$${\dot{x}}_{C2}\left(t\right)=\left(f_{2}-f_{C}\right)F_{C2}\left(t\right)-\delta x_{C2}\left(t\right)$$ (4)

In the above formula, $f_{1}F_{C1}\left(t\right)$ represents the reputation gained by the delivery platform from the centralized scheduling of shared delivery boxes. $f_{C}F_{C1}\left(t\right)$ indicates the impact on the delivery platform due to lower response speeds. $\delta x_{C1}\left(t\right)$ represents the decline in the delivery platform’s reputation. $f_{2}F_{C2}\left(t\right)$ represents the reputation gained by catering enterprises from the centralized scheduling of shared delivery boxes. $f_{C}F_{C2}\left(t\right)$ indicates the impact on catering enterprises due to lower response speeds. $\delta x_{C2}\left(t\right)$ represents the decline in the reputation of catering enterprises.

In the process of efficiently coordinating shared takeaway containers between delivery platforms and food service businesses, a decentralized scheduling model distributes the scheduling and management responsibilities across multiple independent units or locations. While decentralized scheduling offers advantages in flexibility and responsiveness, it also comes with higher management complexity and costs. This model allows for the rapid adjustment of container supply and scheduling in response to changes in demand at different locations and times, thus avoiding the delayed responses that can result from centralized management. Such flexibility enables food service businesses to better cope with sudden order surges or special event demands. Decentralized scheduling permits each food service business or service point to customize container management and service plans according to their specific needs and those of their customers, which can enhance customer satisfaction and increase the business’s market competitiveness. This model also allows for the flexible adjustment of operational strategies based on regional differences, customer preferences, and market environments, making it more adaptable to diverse market conditions, particularly in markets where diverse demands are prominent. As each service point can independently make decisions and innovate, the decentralized scheduling model fosters the emergence of new management methods and service models, driving the development and progress of the entire industry [32].

Decentralized scheduling requires efficient coordination and communication between various service points to ensure the smooth operation of the entire system. However, due to the independent operation of each party, poor communication or inadequate coordination may lead to resource waste and reduced efficiency [9]. Since each service point can make independent decisions based on its own situation, it is challenging to achieve standardized management across the entire system, which may result in inconsistent container quality and service levels, thereby negatively impacting the user experience. Decentralized management often necessitates greater investment in human resources, facilities, and equipment to support the independent operation of multiple service points, leading to increased overall operational costs. Without the economies of scale that centralized management provides, individual service points may need to invest repeatedly in facilities and technology, further adding to the financial burden on businesses.

If takeaway platform and catering companies dispatch shared takeout boxes through the mode of decentralized scheduling, then their social welfare function can be expressed as:


$$J_{D1}=\int_{0}^{\infty } [\left(a_{1}-a_{D}\right)F_{D1}\left(t\right)-\frac{c_{1}}{2}F_{D1}^{2}\left(t\right)\left.+lx_{D1}\left(t\right)\right]\;e^{-\rho t}dt$$ (5)

$$J_{D2}=\int_{0}^{\infty } [a_{2}F_{D2}\left(t\right)-\frac{\left(c_{2}+c_{D}\right)}{2}F_{D2}^{2}\left(t\right)\left.+lx_{D2}\left(t\right)\right]\;e^{-\rho t}dt$$ (6)

In the above formula, $a_{1}F_{D1}\left(t\right)$ indicates the revenue gained from decentralized scheduling of shared takeout containers by takeout platforms. $a_{D}F_{D1}\left(t\right)$ represents the decrease in revenue due to the increased management difficulty of the takeaway platform under the decentralized dispatching model. $\frac{c_{1}}{2}F_{D1}^{2}\left(t\right)$ indicates the costs incurred by the takeaway platform in decentralizing the dispatch of shared takeaway containers. $lx_{D1}\left(t\right)$ indicates the positive impact on the reputation of the takeaway platform. $a_{2}F_{D2}\left(t\right)$ represents the revenue gained by catering enterprises from decentralized dispatch of shared takeaway meal boxes. $\frac{c_{2}}{2}F_{D2}^{2}\left(t\right)$ indicates the cost to catering companies of decentralized scheduling of shared takeaway meal boxes. $\frac{c_{D}}{2}F_{D2}^{2}\left(t\right)$ indicates the increased cost to the catering company as a result of decentralized scheduling. $lx_{D2}\left(t\right)$ indicates the positive impact on the reputation of the catering company.

The change in the reputation of takeaway platform and catering companies under the mode of decentralized scheduling can be expressed as:


$${\dot{x}}_{D1}\left(t\right)=\left(f_{1}+f_{D}\right)F_{D1}\left(t\right)-\delta x_{D1}\left(t\right)$$ (7)

$${\dot{x}}_{D2}\left(t\right)=\left(f_{2}+f_{D}\right)F_{D2}\left(t\right)-\delta x_{D2}\left(t\right)$$ (8)

In the above formula, $f_{1}F_{D1}\left(t\right)$ represents the reputation gained by the delivery platform from the decentralized scheduling of shared delivery boxes. $f_{D}F_{D1}\left(t\right)$ indicates the impact on the delivery platform’s reputation due to improvements in flexibility and response speed. $\delta x_{C1}\left(t\right)$ represents the decline in the delivery platform’s reputation. $f_{2}F_{D2}\left(t\right)$ represents the reputation gained by catering enterprises from the decentralized scheduling of shared delivery boxes. $f_{D}F_{D2}\left(t\right)$ indicates the impact on catering enterprises due to improvements in flexibility and response speed. $\delta x_{C2}\left(t\right)$ represents the decline in the reputation of catering enterprises.

In the process of efficiently coordinating shared takeaway containers between delivery platforms and food service businesses, a demand-based scheduling model leverages real-time demand data for resource scheduling and allocation. This model primarily relies on advanced technological methods to achieve precise matching of takeaway container supply and demand, although it involves significant technical requirements and cost investments. By monitoring order data and demand changes in real time, the demand-based scheduling model can accurately predict and allocate container needs, preventing the idle and waste of excess containers, thus improving resource utilization efficiency. Through big data analysis and artificial intelligence, the demand-based scheduling model can deeply analyze user behavior, consumption habits, and market trends, providing a scientific basis for decision-making [32]. This approach helps food service businesses to better formulate operational strategies and optimize service processes.

The demand-based scheduling model relies on highly complex technological methods, such as big data analysis, real-time monitoring, artificial intelligence, and the Internet of Things. This necessitates extensive software development, technology integration, and system maintenance, placing significant demands on the technical team. The construction and maintenance of a demand-based scheduling system require substantial initial investments, including technology development, hardware equipment, data storage, and network infrastructure. For small to medium-sized food service businesses or platforms, this may pose considerable financial pressure. In addition to the initial investment, the demand-based scheduling model also incurs ongoing operational maintenance costs, such as system upgrades, server maintenance, and data security management, further increasing the financial burden on businesses [33]. The model also necessitates the collection and analysis of large amounts of user data, which raises heightened concerns about data privacy and protection. Inadequate data security measures could lead to user privacy breaches, resulting in legal and ethical risks.

If takeaway platform and catering companies dispatch shared takeout boxes through the mode of demand scheduling, then their social welfare function can be expressed as:


$$J_{B1}=\int_{0}^{\infty } [\left(a_{1}+a_{B}\right)F_{B1}\left(t\right)-\frac{c_{1}+c_{B}}{2}F_{B1}^{2}\left(t\right)\left.+lx_{B1}\left(t\right)\right]\;e^{-\rho t}dt$$ (9)

$$J_{B2}=\int_{0}^{\infty } [a_{2}F_{B2}\left(t\right)-\frac{c_{2}}{2}F_{B2}^{2}\left(t\right)\left.+lx_{B2}\left(t\right)\right]\;e^{-\rho t}dt$$ (10)

In the above formula, $a_{1}F_{B1}\left(t\right)$ represents the benefits obtained by the delivery platform from demand scheduling of shared delivery boxes. $a_{B}F_{B1}\left(t\right)$ indicates the increased benefits due to technological improvements under the demand scheduling model. $\frac{c_{1}}{2}F_{B1}^{2}\left(t\right)$ represents the costs incurred by the delivery platform for demand scheduling of shared delivery boxes. $\frac{c_{B}}{2}F_{B1}^{2}\left(t\right)$ indicates the costs of technological investment under the demand scheduling model. $lx_{B1}\left(t\right)$ represents the positive impact on the delivery platform’s reputation. $a_{2}F_{B2}\left(t\right)$ represents the benefits obtained by catering enterprises from demand scheduling of shared delivery boxes. $\frac{c_{2}}{2}F_{B2}^{2}\left(t\right)$ represents the costs incurred by catering enterprises for demand scheduling of shared delivery boxes. $lx_{B2}\left(t\right)$ represents the positive impact on the reputation of catering enterprises.

The change in the reputation of takeaway platform and catering companies under the mode of demand scheduling can be expressed as:


$${\dot{x}}_{B1}\left(t\right)=f_{1}F_{B1}\left(t\right)-\delta x_{B1}\left(t\right)$$ (11)

$${\dot{x}}_{B2}\left(t\right)=f_{2}F_{B2}\left(t\right)-\delta x_{B2}\left(t\right)$$ (12)

In the above formula, $f_{1}F_{B1}\left(t\right)$ denotes the reputation gained by the takeaway platform’s need to dispatch shared takeaway lunch boxes. $\delta x_{B1}\left(t\right)$ denotes the decay of the takeout platform’s reputation. $f_{2}F_{B2}\left(t\right)$ denotes the attenuation of the reputation gained by the demand scheduling of shared takeaway lunchboxes by catering companies. $\delta x_{B2}\left(t\right)$ denotes the attenuation of the reputation of the catering business.

## 3. Results

In the differential game, the takeaway platform and catering companies in the process of dispatching shared takeout boxes are not only affected by control variables and parameters, but also change over time. In order to better calculate the control benefits and social benefits, the HJB formula is used. The HJB formula is a partial differential equation, which is the core of optimal control.

### 3.1 HJB formula

Under the mode of centralized scheduling, the HJB equation of the social welfare function of the takeaway platform and catering companies are:


$$\rho V_{C1}=\underset{F_{C1}\left(t\right)}{\max} \{ [\left(a_{1}+a_{C}\right)F_{C1}\left(t\right)-\frac{c_{1}}{2}F_{C1}^{2}\left(t\right)+\left.lx_{C1}\left(t\right)\right]+\left.\frac{\partial V_{C1}}{\partial x_{C1}}\left[\left(f_{1}-f_{C}\right)F_{C1}\left(t\right)-\delta x_{C1}\left(t\right)\right]\right\}$$ (13)

$$\rho V_{C2}=\underset{F_{C2}\left(t\right)}{\max} \{ [a_{2}F_{C2}\left(t\right)-\frac{c_{2}}{2\ln\left(\text{e}+b_{C}\right)}F_{C2}^{2}\left(t\right)+\left.lx_{C2}\left(t\right)\right]+\left.\frac{\partial V_{C2}}{\partial x_{C2}}\left[\left(f_{2}-f_{C}\right)F_{C2}\left(t\right)-\delta x_{C2}\left(t\right)\right]\right\}$$ (14)

Under the mode of decentralized scheduling, the HJB equation of the social welfare function of the takeaway platform and catering companies are:


$$\rho V_{D1}=\underset{F_{D1}\left(t\right)}{\max} \{ [\left(a_{1}-a_{D}\right)F_{D1}\left(t\right)-\frac{c_{1}}{2}F_{D1}^{2}\left(t\right)+\left.lx_{D1}\left(t\right)\right]+\left.\frac{\partial V_{D1}}{\partial x_{D1}}\left[\left(f_{1}+f_{D}\right)F_{D1}\left(t\right)-\delta x_{D1}\left(t\right)\right]\right\}$$ (15)

$$\rho V_{D2}=\underset{F_{D2}\left(t\right)}{\max} \{ [a_{2}F_{D2}\left(t\right)-\frac{\left(c_{2}+c_{D}\right)}{2}F_{D2}^{2}\left(t\right)+\left.lx_{D2}\left(t\right)\right]+\left.\frac{\partial V_{D2}}{\partial x_{D2}}\left[\left(f_{2}+f_{D}\right)F_{D2}\left(t\right)-\delta x_{D2}\left(t\right)\right]\right\}$$ (16)

Under the mode of demand scheduling, the HJB equation of the social welfare function of the takeaway platform and catering companies are:


$$\rho V_{B1}=\underset{F_{B1}\left(t\right)}{\max} \{ [\left(a_{1}+a_{B}\right)F_{B1}\left(t\right)-\frac{c_{1}+c_{B}}{2}F_{B1}^{2}\left(t\right)+\left.lx_{B1}\left(t\right)\right]+\left.\frac{\partial V_{B1}}{\partial x_{B1}}\left[f_{1}F_{B1}\left(t\right)-\delta x_{B1}\left(t\right)\right]\right\}$$ (17)

$$\rho V_{B2}=\underset{F_{B2}\left(t\right)}{\max} \{ [a_{2}F_{B2}\left(t\right)-\frac{c_{2}}{2}F_{B2}^{2}\left(t\right)+\left.lx_{F2}\left(t\right)\right]+\left.\frac{\partial V_{B2}}{\partial x_{B2}}\left[f_{2}F_{B2}\left(t\right)-\delta x_{B2}\left(t\right)\right]\right\}$$ (18)

### 3.2 Result of equilibrium

Proposition 1: Under the mode of centralized scheduling, the efforts to dispatch takeaway lunch boxes and social benefits of takeaway platform and catering companies are respectively (the specific solving procedure is shown in S1 File):


$$F_{C1}^{*}\left(t\right)=\frac{a_{1}+a_{C}}{c_{1}}+\frac{f_{1}-f_{C}}{c_{1}}\frac{l}{\rho +\delta }$$ (19)

$$F_{C2}^{*}\left(t\right)=\left(\frac{a_{2}}{c_{2}}+\frac{f_{2}-f_{C}}{c_{2}}\frac{l}{\rho +\delta }\right)\ln\left(\text{e}+b_{C}\right)$$ (20)

$$\begin{array}{l} V_{C1}^{*}=\frac{l}{\rho +\delta }x_{C1}+\frac{1}{\rho }\left(a_{1}+a_{C}\right)\left(\frac{a_{1}+a_{C}}{c_{1}}+\frac{f_{1}-f_{C}}{c_{1}}\frac{l}{\rho +\delta }\right)-\frac{c_{1}}{2}\frac{1}{\rho }{\left(\frac{a_{1}+a_{C}}{c_{1}}+\frac{f_{1}-f_{C}}{c_{1}}\frac{l}{\rho +\delta }\right)}^{2}\\ \;+\frac{l}{\rho +\delta }\frac{1}{\rho }\left(f_{1}-f_{C}\right)\left(\frac{a_{1}+a_{C}}{c_{1}}+\frac{f_{1}-f_{C}}{c_{1}}\frac{l}{\rho +\delta }\right) \end{array}$$ (21)

$$\begin{array}{l} V_{C2}^{\text{*}}=\frac{1}{\rho }a_{2}\left(\frac{a_{2}}{c_{2}}+\frac{f_{2}-f_{C}}{c_{2}}\frac{l}{\rho +\delta }\right)\ln\left(\text{e}+b_{C}\right)-\frac{1}{\rho }\frac{c_{2}}{2\ln\left(\text{e}+b_{C}\right)}{\left(\frac{a_{2}}{c_{2}}+\frac{f_{2}-f_{C}}{c_{2}}\frac{l}{\rho +\delta }\right)}^{2}{\left[\ln\left(\text{e}+b_{C}\right)\right]}^{2}\\ \;+\frac{1}{\rho }\frac{l}{\rho +\delta }\left(f_{2}-f_{C}\right)\left(\frac{a_{2}}{c_{2}}+\frac{f_{2}-f_{C}}{c_{2}}\frac{l}{\rho +\delta }\right)\ln\left(\text{e}+b_{C}\right)+\frac{l}{\rho +\delta }x_{C2} \end{array}$$ (22)

Conclusion 1: The greater the adverse impact caused by a decrease in response speed, the less effort food delivery platforms or restaurant businesses will put into coordinating the use of shared takeaway containers. Conversely, the better the cost control by restaurant businesses, the more effort they will invest in coordinating the use of shared takeaway containers.

Proposition 2: Under the mode of decentralized scheduling, the efforts to dispatch takeaway lunch boxes and social benefits of takeaway platform and catering companies are respectively (the specific solving procedure is shown in S2 File):


$$F_{D1}^{*}\left(t\right)=\frac{a_{1}-a_{D}}{c_{1}}+\frac{f_{1}+f_{D}}{c_{1}}\frac{l}{\rho +\delta }$$ (23)

$$F_{D2}^{\text{*}}\left(t\right)=\frac{a_{2}}{c_{2}+c_{D}}+\frac{f_{2}+f_{D}}{c_{2}+c_{D}}\frac{l}{\rho +\delta }$$ (24)

$$\begin{array}{l} V_{D1}^{*}=\frac{l}{\rho +\delta }x_{D1}+\frac{1}{\rho }\left(a_{1}-a_{D}\right)\left(\frac{a_{1}-a_{D}}{c_{1}}+\frac{f_{1}+f_{D}}{c_{1}}\frac{l}{\rho +\delta }\right)-\frac{c_{1}}{2}\frac{1}{\rho }{\left(\frac{a_{1}-a_{D}}{c_{1}}+\frac{f_{1}+f_{D}}{c_{1}}\frac{l}{\rho +\delta }\right)}^{2}\\ \;+\frac{1}{\rho }\frac{l}{\rho +\delta }\left(f_{1}+f_{D}\right)\left(\frac{a_{1}-a_{D}}{c_{1}}+\frac{f_{1}+f_{D}}{c_{1}}\frac{l}{\rho +\delta }\right) \end{array}$$ (25)

$$\begin{array}{l} V_{D2}^{\text{*}}=\frac{l}{\rho +\delta }x_{D2}+\frac{1}{\rho }a_{2}\left(\frac{a_{2}}{c_{2}+c_{D}}+\frac{f_{2}+f_{D}}{c_{2}+c_{D}}\frac{l}{\rho +\delta }\right)-\frac{\left(c_{2}+c_{D}\right)}{2}\frac{1}{\rho }{\left(\frac{a_{2}}{c_{2}+c_{D}}+\frac{f_{2}+f_{D}}{c_{2}+c_{D}}\frac{l}{\rho +\delta }\right)}^{2}\\ \;+\frac{l}{\rho +\delta }\frac{1}{\rho }\left(f_{2}+f_{D}\right)\left(\frac{a_{2}}{c_{2}+c_{D}}+\frac{f_{2}+f_{D}}{c_{2}+c_{D}}\frac{l}{\rho +\delta }\right) \end{array}$$ (26)

Conclusion 2: The greater the positive impact of increased flexibility and response speed on reputation, the more effort food delivery platforms and restaurant businesses will invest in coordinating the use of shared takeaway containers; conversely, the greater the cost increase for restaurant businesses due to decentralized scheduling, the less effort they will put into coordinating the use of shared takeaway containers.

Proposition 3: Under the mode of demand scheduling, the efforts to dispatch takeaway lunch boxes and social benefits of takeaway platform and catering companies are respectively (the specific solving procedure is shown in S3 File):


$$F_{B1}^{\text{*}}\left(t\right)\text{=}\frac{a_{1}+a_{B}}{c_{1}+c_{B}}+\frac{f_{1}}{c_{1}+c_{B}}\frac{l}{\rho +\delta }$$ (27)

$$F_{B2}^{\text{*}}\left(t\right)\text{=}\frac{a_{2}}{c_{2}}+\frac{f_{2}}{c_{2}}\frac{l}{\rho +\delta }$$ (28)

$$\begin{array}{l} V_{B1}^{*}=\frac{l}{\rho +\delta }x_{B1}+\frac{1}{\rho }\left(a_{1}+a_{B}\right)\left(\frac{a_{1}+a_{B}}{c_{1}+c_{B}}+\frac{f_{1}}{c_{1}+c_{B}}\frac{l}{\rho +\delta }\right)-\frac{c_{1}+c_{B}}{2}\frac{1}{\rho }{\left(\frac{a_{1}+a_{B}}{c_{1}+c_{B}}+\frac{f_{1}}{c_{1}+c_{B}}\frac{l}{\rho +\delta }\right)}^{2}\\ \;+\frac{l}{\rho +\delta }\frac{1}{\rho }f_{1}\left(\frac{a_{1}+a_{B}}{c_{1}+c_{B}}+\frac{f_{1}}{c_{1}+c_{B}}\frac{l}{\rho +\delta }\right) \end{array}$$ (29)

$$V_{B2}^{\text{*}}=\frac{l}{\rho +\delta }x_{B2}+\frac{1}{\rho }a_{2}\left(\frac{a_{2}}{c_{2}}+\frac{f_{2}}{c_{2}}\frac{l}{\rho +\delta }\right)-\frac{c_{2}}{2}\frac{1}{\rho }{\left(\frac{a_{2}}{c_{2}}+\frac{f_{2}}{c_{2}}\frac{l}{\rho +\delta }\right)}^{2}+\frac{l}{\rho +\delta }\frac{1}{\rho }f_{2}\left(\frac{a_{2}}{c_{2}}+\frac{f_{2}}{c_{2}}\frac{l}{\rho +\delta }\right)$$ (30)

Conclusion 3: The greater the return on investment in technology, the more effort food delivery platforms will invest in coordinating the use of shared takeaway containers; conversely, the higher the cost of technological investment, the less effort food delivery platforms will put into coordinating the use of shared takeaway containers.

### 3.3 Numerical analysis

In order to describe the changes in the social welfare functions of takeaway platform and catering companies in the process of dispatching takeaway lunch boxes, this paper adopts the method of numerical analysis. The following assumptions are made for relevant parameters.

The primary benefits of integrating resources for food delivery platforms include the following aspects. First, cost reduction through economies of scale. By centralizing procurement and distribution, the cost per food box is reduced. Second, supply chain optimization. Improving procurement and distribution efficiency lowers logistics and management costs. Third, enhanced user experience. Increasing user stickiness and satisfaction boosts the overall business volume and repurchase rate of the platform. The revenue that food delivery platforms obtain from delivering food boxes mainly comes from delivery fees and service fees. Based on experience and industry analysis, it can be assumed that the benefits from integrated resources account for approximately 10% to 30% of the revenue from direct delivery. This ratio specifically depends on the platform’s operational capabilities, market environment, and the specific implementation of resource integration. For convenience, this paper assumes a median value of approximately 20%. Therefore, this paper hypothesizes that the gains *a*_*C*_ from resource consolidation by takeaway platform is 0.2*a*_1_.

The ratio of “revenue reduction due to increased management difficulty on the platform” to “revenue generated from delivering food boxes” is influenced by several factors. Firstly, the increase in management complexity leads to more intricate resource allocation and higher coordination costs. Sharing food boxes among multiple catering businesses complicates scheduling and management, necessitating more efficient systems and increased manual intervention. This process requires more communication and coordination, which raises management and operational costs [23]. Secondly, the increase in management complexity may lead to increased customer service pressure. As shared food boxes become more widely used, users may encounter more service issues, resulting in more complaints and disputes that require additional customer service resources to resolve. Thirdly, increased management complexity can potentially lead to a decline in operational efficiency. This complexity requires managing a greater number of suppliers and distribution chains, increasing the intricacy of supply chain management. Based on industry experience and the increase in management costs, the proportion of revenue reduction due to increased management difficulty is generally estimated to be between 5% and 15% of total revenue. For convenience, this paper assumes a median value of approximately 10%. Therefore, this paper hypothesizes that reduced revenue *a*_*D*_ due to increased difficulty in managing takeaway platforms is 0.1*a*_1_.

In the process of dispatching shared food boxes between food delivery platforms and catering businesses, the ratio of “return on increased technology investment” to “revenue generated from delivering food boxes” varies depending on factors such as the scale of the platform’s technology investment, the effectiveness of technology implementation, and the market environment. The returns on technology investment are primarily reflected in the following areas. Firstly, increased operational efficiency. By utilizing automated scheduling systems and intelligent distribution algorithms, delivery efficiency can be improved, and operational costs can be reduced. Secondly, improved service quality. Through data analysis and predictive technologies, user needs can be better met, enhancing customer satisfaction and retention. Thirdly, cost reduction. Technological means can optimize supply chain management, reduce waste, and improve resource utilization. Fourthly, expanded market capabilities. Technological innovation can rapidly extend service areas and capture greater market share. Based on industry experience, the return on technology investment as a proportion of total revenue can range from 10% to 25%. For convenience, this paper assumes a median value of approximately 17%. Therefore, this paper hypothesizes that increased returns *a*_*B*_ from investing in technology is 0.17*a*_1_.

The “impact of reduced response speed by food delivery platforms or catering businesses” is generally considered greater than the “impact of improving flexibility and response speed on reputation” for the following reasons. Firstly, the negative impact is direct and apparent. The decrease in response speed has an immediate and obvious negative effect, as users will instantly perceive the decline in service quality, potentially leading to immediate order cancellations and customer attrition. In contrast, the positive impact of improving response speed on reputation, although important, requires some time to accumulate and manifest [22]. Secondly, user expectation management. Users typically expect timely and efficient service from food delivery platforms. If the platform or catering business fails to meet this basic expectation, users will quickly become dissatisfied and seek alternative options. This negative impact strikes the platform swiftly and profoundly. Thirdly, market competition pressure. In a highly competitive market, response speed is a fundamental competitive advantage for platforms and catering businesses. If rapid response cannot be ensured, it is easy for competitors to capture market share, leading to long-term brand and market losses. Therefore, this paper hypothesizes that the impact factor *f*_*C*_ of lower responsiveness on takeout platforms or caterers is 0.3*f*; the reputational impact *f*_*D*_ of improvements in flexibility and responsiveness 0.2*f.*

“Decentralized scheduling leading to increased costs for catering businesses” and “technology investment costs” depend on various factors. Firstly, the increased costs of decentralized scheduling typically manifest in the short term, directly and significantly affecting the daily operations of businesses. In contrast, technology investment costs may be high initially, but the efficiency improvements and cost savings brought by technology investment become evident over time, potentially offering more returns in the long run. Secondly, the operational costs of decentralized scheduling are primarily ongoing operational expenses, such as labor, logistics, and management, which occur daily [11]. At the same time, the initial investment costs for technology are significant, but subsequent expenses are mainly for maintenance and upgrades, making them more one-time and fixed compared to the operational costs of decentralized scheduling. Thirdly, decentralized scheduling is primarily to meet the demands of different platforms, with limited benefits mainly aimed at adapting to market competition. Conversely, technology investment can significantly improve operational efficiency, reduce costs, and enhance competitiveness, making its long-term benefits more apparent. In summary, technology investment costs are greater than the increased costs for catering businesses due to decentralized scheduling, as technology investment requires substantial initial expenditure and ongoing maintenance and upgrade costs. Therefore, this paper hypothesizes that the cost *c*_*B*_ of investing in technology is 0.5*c*; the increased costs *c*_*D*_ to catering companies resulting from decentralized scheduling is 0.4*c.*

Meanwhile, the paper makes the following assumptions about other variables that do not affect the results. The reputation *f*_1_, *f*_2_ that the takeaway platform or catering companies gain by dispatching takeaway lunch boxes at a unit level is 2; the positive effects *l* of reputation is 1; the discount rate *ρ* that occurs over time is 0.9; impact factor *b*_*C*_ of cost control on catering companies is 0.5e; decay *δ* of reputation is 0.1. Also in this paper, it is assumed to be in a unit state, i.e., the state variable is 1.

When the cost *c*_1_ to the takeaway platform of dispatching takeaway lunch boxes at a unit level is 1, this article can calculate the social benefits of takeaway platform:


$$V_{C1}^{*}=1+0.556{\left(1.2a_{1}+1.4\right)}^{2}$$ (31)

$$V_{D1}^{*}=1+0.556{\left(0.9a_{1}+2.4\right)}^{2}$$ (32)

$$V_{B1}^{*}=1+0.83{\left(0.78a_{1}+1.333\right)}^{2}$$ (33)

**Fig 2:**
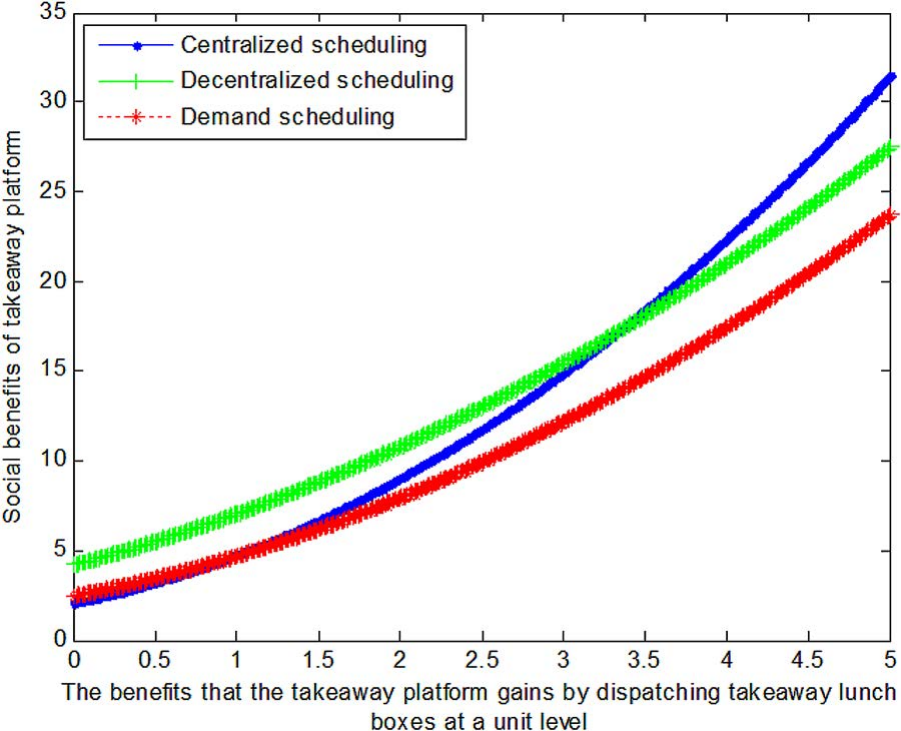
Impact of benefits of takeaway platform on social welfare.

The following graph (named Fig 2) can also be produced:

When the cost *c*_1_ to the takeaway platform of dispatching takeaway lunch boxes at a unit level is 3, this article can calculate the social benefits of takeaway platform:


$$V_{C1}^{*}=1+1.667{\left(0.4a_{1}+0.47\right)}^{2}$$ (34)

$$V_{D1}^{*}=1+1.67{\left(0.3a_{1}+0.8\right)}^{2}$$ (35)

$$V_{B1}^{*}=1+2.5{\left(0.26a_{1}+0.44\right)}^{2}$$ (36)

**Fig 3:**
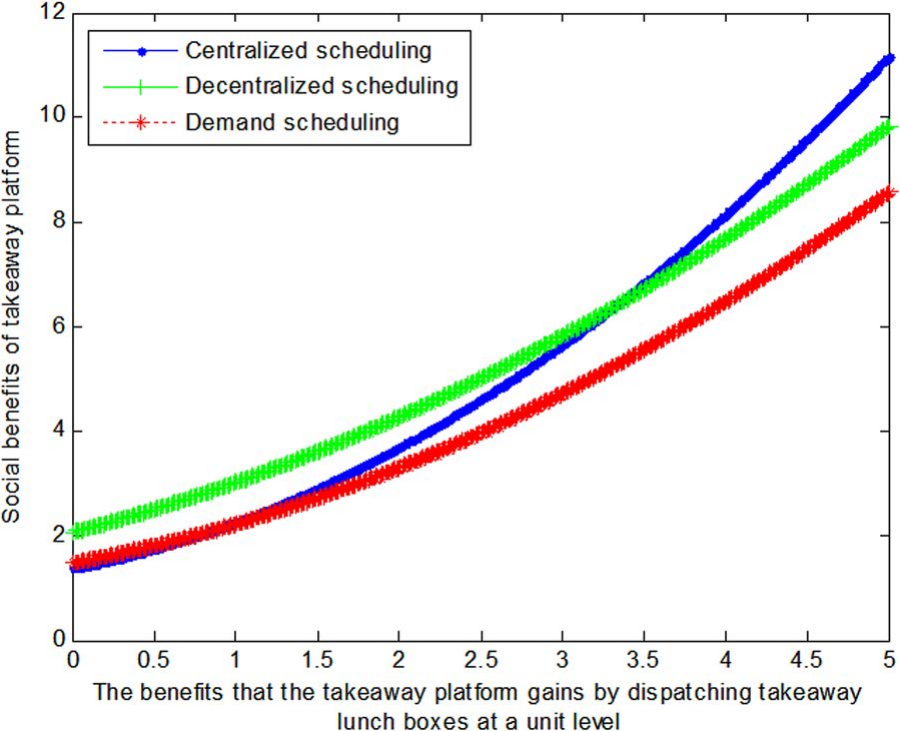
Impact of benefits of takeaway platform on social welfare.

The following graph (named Fig 3) can also be produced:

Conclusion 4: When the benefits gained from coordinating takeaway containers are relatively small, decentralized scheduling can maximize the benefits for food delivery platforms; conversely, when the benefits gained from coordinating takeaway containers are substantial, centralized scheduling can maximize the benefits for food delivery platforms.

When the cost *c*_2_ to the catering companies of dispatching takeaway lunch boxes at a unit level is 1, this article can calculate the social benefits of catering companies:


$$V_{C2}^{\text{*}}=0.78{\left(a_{2}+1.4\right)}^{2}+1$$ (37)

$$V_{D2}^{\text{*}}=1+0.777{\left(0.72a_{2}+1.71\right)}^{2}$$ (38)

$$V_{B2}^{\text{*}}=1+0.556{\left(a_{2}+2\right)}^{2}$$ (39)

**Fig 4:**
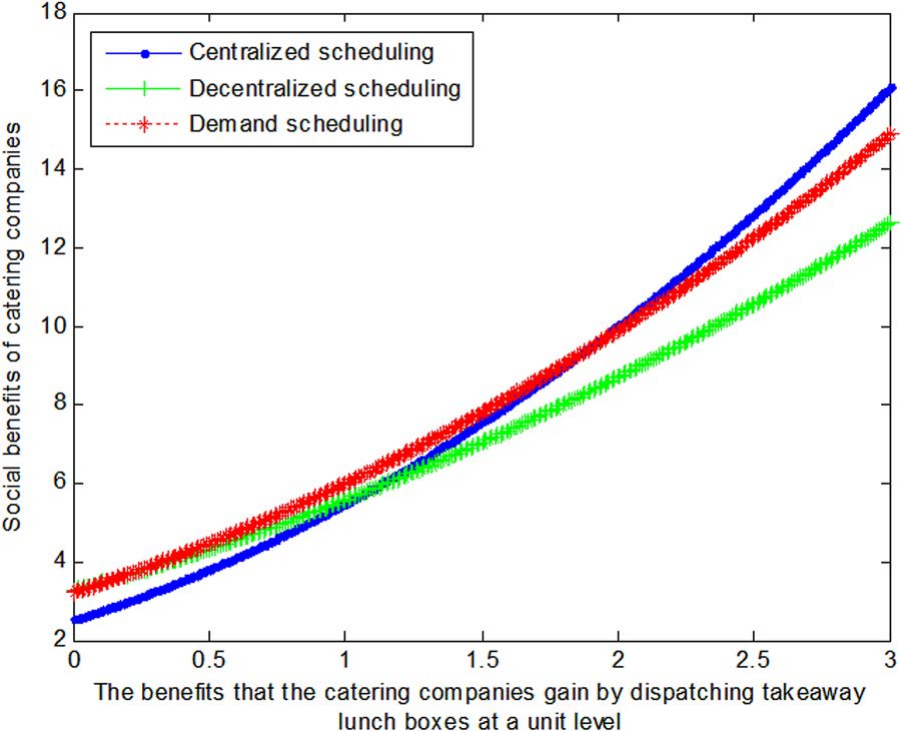
Impact of benefits of catering companies on social welfare.

The following graph (named Fig 4) can also be produced:

Conclusion 5: If the cost of coordinating takeaway containers is relatively low, demand-based scheduling can maximize the benefits for restaurant businesses when the gains from coordinating takeaway containers are small; however, when the gains from coordinating takeaway containers are substantial, centralized scheduling can maximize the benefits for restaurant businesses.

When the cost *c*_2_ to the catering companies of dispatching takeaway lunch boxes at a unit level is 3, this article can calculate the social benefits of catering companies:


$$V_{C2}^{\text{*}}=2.35{\left(0.333a_{2}+0.47\right)}^{2}+1$$ (40)

$$V_{D2}^{\text{*}}=1+1.833{\left(0.30a_{2}+0.73\right)}^{2}$$ (41)

$$V_{B2}^{\text{*}}=1+1.67{\left(0.333a_{2}+0.667\right)}^{2}$$ (42)

**Fig 5:**
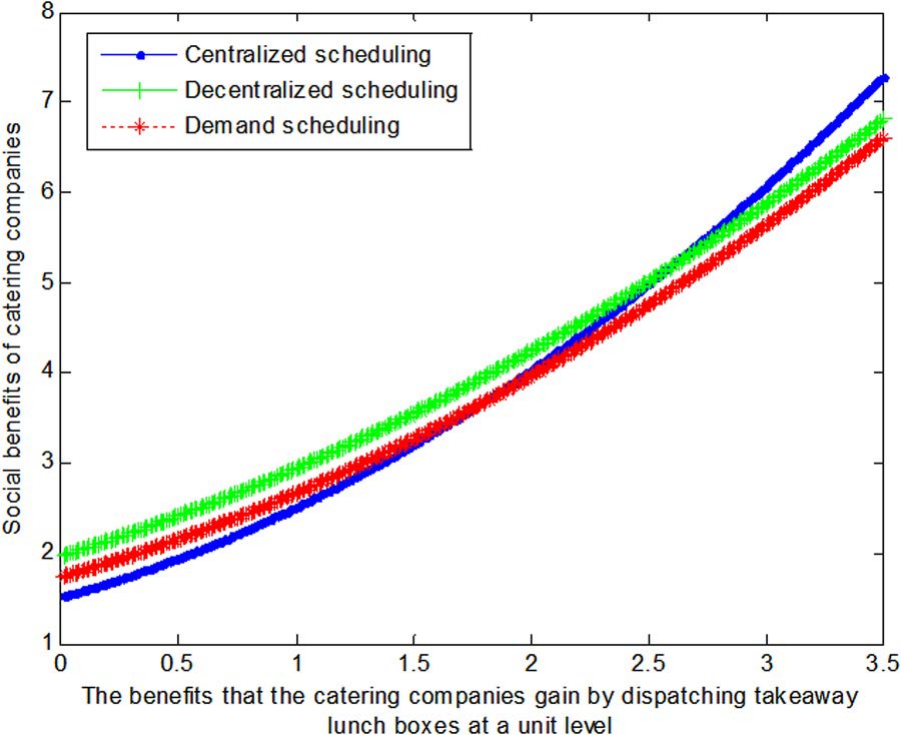
Impact of benefits of catering companies on social welfare.

The following graph (named Fig 5) can also be produced:

Conclusion 6: If the cost of coordinating takeaway containers is relatively high, decentralized scheduling can maximize the benefits for restaurant businesses when the gains from coordinating takeaway containers are small; however, when the gains from coordinating takeaway containers are substantial, centralized scheduling can maximize the benefits for restaurant businesses.

## 4. Discussion

A key factor in food delivery services is fast delivery. In Conclusion 1, if the response speed decreases, it means that the delivery time is extended, which will affect customer satisfaction. Customers may become dissatisfied with the long waiting time and may even switch to other more efficient service providers. Managing and coordinating the scheduling of shared delivery boxes incurs certain costs. If the response speed decreases, the delivery platform and restaurants may need to invest more resources in managing this sharing mechanism, including logistics arrangements and real-time monitoring, which will increase operating costs [24]. Shared delivery boxes are intended to improve resource utilization and reduce environmental pollution. However, if the response speed decreases, untimely turnover of boxes may lead to shortages or backlogs, reducing overall operational efficiency. In the highly competitive food delivery market, fast response and efficient delivery are core competencies. If the scheduling of shared boxes affects the response speed, delivery platforms or restaurants may lose their competitive edge, affecting market share and profitability. Therefore, when the adverse effects of reduced response speed become more significant, delivery platforms or restaurants may find it not worth investing excessive resources and effort into scheduling shared delivery boxes, as this may bring more negative effects rather than the expected positive benefits.

According to Conclusion 1, the better the cost control of a catering enterprise, the greater the effort it will put into scheduling shared delivery boxes. This is primarily due to the following reasons. Shared delivery boxes are designed to reduce the use of disposable containers, thereby lowering procurement and handling costs for businesses. If a catering enterprise excels in cost control, it is more likely to seek various methods to further reduce expenses. Scheduling shared delivery boxes is an effective way to help them save more costs. Good cost control means that the catering enterprise can utilize resources more efficiently. A shared delivery box system can optimize logistics and storage costs through centralized management and resource allocation. This optimization is particularly attractive to enterprises with good cost control because they are accustomed to meticulous management and efficient operations [23]. As environmental awareness in society increases, catering enterprises with good cost control are also more likely to focus on environmental protection and social responsibility. Shared delivery boxes help reduce the use of disposable plastic products, thereby reducing environmental pollution. Such environmental measures not only help establish a positive corporate image but also bring long-term economic benefits. Effective cost control enables catering enterprises to be more competitive in pricing. If they can further reduce costs through shared delivery boxes, they can offer more attractive prices, thus attracting more customers and increasing market share. Enterprises with good cost control typically possess strong management and operational capabilities, making it easier for them to collaborate with delivery platforms and successfully implement a shared delivery box scheduling system. This synergy further incentivizes enterprises to put more effort into effectively scheduling shared delivery boxes. Therefore, the better a catering enterprise’s cost control, the more willing and capable it will be to invest resources and effort into scheduling shared delivery boxes, as this aligns with their cost control strategies and brings additional economic and social benefits.

According to Conclusion 2, the greater the positive impact of increased flexibility and response speed on reputation, the more effort delivery platforms and catering enterprises will put into scheduling shared delivery boxes. This assertion can be explained from the following perspectives. First, increasing flexibility and response speed means customers can receive their deliveries faster, significantly enhancing their satisfaction and experience. Satisfied customers are more likely to become loyal patrons and provide positive reviews on social media and through word-of-mouth, benefiting the platform and enterprises. Second, in the highly competitive food delivery market, flexibility and response speed are crucial competitive advantages. Companies that can quickly respond to orders and provide flexible services are more likely to stand out in the market, attracting more customers. This competitive edge directly affects market share and profitability. Third, improvements in flexibility and response speed help shape the brand image of a company [11]. Companies perceived as efficient and customer-friendly usually enjoy better public reputations, which is vital for long-term business development. A good brand image can lead to more business opportunities and partnerships. Fourth, scheduling shared delivery boxes helps improve resource utilization, reducing unnecessary waste and expenses. If delivery platforms and catering enterprises can efficiently schedule shared boxes, they can better manage inventory and logistics, further improving overall operational efficiency. This enhancement not only reduces operating costs but also increases the company’s resilience [24]. Therefore, when the beneficial impact of increased flexibility and response speed on a company’s reputation is greater, delivery platforms and catering enterprises will work harder to schedule shared delivery boxes to ensure fast and efficient service. This not only boosts customer satisfaction and market competitiveness but also strengthens the company’s brand image and operational efficiency, ultimately leading to greater business success and social recognition.

The greater the extent to which decentralized scheduling increases the costs for catering enterprises, the less effort these enterprises will put into scheduling shared delivery boxes. This is the conclusion derived from Conclusion 2. This assertion can be explained from the following perspectives. First, decentralized scheduling means that the management and allocation of delivery boxes need to occur at multiple locations and times, increasing operational complexity. Catering enterprises need to invest more human and material resources to coordinate and monitor these scheduling processes, thereby increasing management costs [9]. Second, decentralized scheduling may result in more delivery routes and schedules, requiring more transportation resources. This leads to higher logistics costs, as more delivery vehicles, fuel, and manpower are needed to cover a wider delivery area and more frequent deliveries. Third, decentralized scheduling makes inventory management of delivery boxes more complex. Enterprises need to maintain a certain number of delivery boxes at multiple locations to ensure an adequate supply at each delivery point. This multi-point inventory management not only increases management difficulty but may also lead to overstocking or shortages, thereby increasing inventory costs. Fourth, in a decentralized scheduling scenario, catering enterprises need to spend more time and resources coordinating the delivery box demands at different locations and times. This coordination work not only increases operational costs but may also reduce scheduling efficiency, affecting overall operational effectiveness [22]. Fifth, decentralized scheduling may lead to frequent transfers of delivery boxes between different locations, increasing resource waste. For example, some delivery boxes may be damaged or lost during transportation or may not be used in time due to improper scheduling, resulting in resource wastage. Therefore, the greater the extent to which decentralized scheduling increases the costs for catering enterprises, the more likely these enterprises are to reduce their efforts in scheduling shared delivery boxes.

According to Conclusion 3, “the greater the return on investment in technology, the greater the effort delivery platforms will put into scheduling shared delivery boxes.” This assertion can be explained from the following perspectives. First, investing in technology can significantly improve the scheduling efficiency of delivery platforms. For example, by using advanced algorithms and artificial intelligence, platforms can more accurately predict demand, optimize delivery routes, and reduce delivery time and costs. When technological investments lead to noticeable efficiency improvements, platforms are more willing to allocate resources and effort to schedule shared delivery boxes, as this can result in higher operational benefits. Second, technological investment can help delivery platforms achieve better resource management and cost control. For instance, through automated inventory management systems, platforms can reduce human errors and waste, increasing the utilization rate of shared delivery boxes. The more significant the cost-saving effects, the more motivated the platforms will be to enhance the scheduling of shared delivery boxes. Third, the application of advanced technology can improve customer experience, such as faster delivery speeds, more accurate delivery time predictions, and better service quality [13]. When the returns on technological investment include significantly increased customer satisfaction, platforms will be more diligent in scheduling shared delivery boxes, as satisfied customers are more likely to become loyal users, enhancing the platform’s market competitiveness and reputation. Fourth, investing in technology allows delivery platforms to better collect and analyze data, enabling them to make more informed decisions. For example, through data analysis, platforms can identify high-demand areas and time periods, optimizing delivery box scheduling and delivery strategies. The stronger the data-driven decision-making capabilities, the greater the platform’s effort in scheduling shared delivery boxes, as data-driven decisions often result in higher operational efficiency. Therefore, the greater the return on investment in technology, the more effort delivery platforms will put into scheduling shared delivery boxes, because the efficiency improvements, cost savings, increased customer satisfaction, enhanced data-driven decision-making capabilities, long-term competitive advantage, and achievement of sustainable development goals brought by technological investment will significantly improve the platform’s operational efficiency and market position.

In the process of effectively scheduling shared delivery boxes by food delivery platforms and catering enterprises, according to Conclusion 3, “the greater the cost of technological investment, the less effort delivery platforms will put into scheduling shared delivery boxes.” This assertion can be explained from the following perspectives. First, increased financial pressure. High technological investment costs can impose significant financial pressure on delivery platforms. This pressure may force platforms to cut budgets in other areas, including resources and personnel dedicated to scheduling shared delivery boxes. With limited resources, platforms may have to prioritize other more urgent investments or operational needs, thus reducing their efforts in shared box scheduling. Second, long investment return cycle. High-cost technological investments typically require a long return cycle. In such cases, delivery platforms may not see immediate returns on their investments, which could lower their enthusiasm for scheduling shared delivery boxes. Platforms might prefer solutions that yield short-term benefits rather than continuing to allocate substantial resources to shared box scheduling. Third, risk assessment. High-cost technological investments come with substantial risks; if the investment fails to deliver the expected returns, platforms could incur significant financial losses [27]. This risk makes platforms more cautious in their decision-making, potentially reducing their attempts and investments in shared box scheduling to avoid further financial risk. Fourth, resource allocation priority. When the cost of technological investment is high, delivery platforms might need to allocate more resources to technology development and maintenance, potentially leading to reduced resources for other projects, including shared box scheduling. In situations of limited resources, platforms will prioritize projects that can deliver direct economic benefits or hold strategic importance. Therefore, when technological investment costs are high, the effort delivery platforms put into scheduling shared delivery boxes diminishes, as factors such as financial pressure, long return cycles, investment risk, changes in resource allocation priority, increased operational complexity, and a short-term benefit orientation all influence the platform’s decisions and resource distribution.

According to Conclusion 4, when the benefits obtained from scheduling delivery boxes are low, decentralized scheduling can maximize the benefits for delivery platforms; conversely, when the benefits are high, centralized scheduling can maximize the benefits. This statement can be explained from the following perspectives. First, when the benefits obtained from scheduling delivery boxes are low, platforms face greater uncertainty and risk. By adopting decentralized scheduling, risk can be distributed across different regions and time points, reducing the impact of any single failure. For example, if demand in one area is low, decentralized scheduling allows resources to be allocated to other areas, minimizing losses. Additionally, when benefits are low, platforms need to expand market coverage as much as possible to increase potential customer touchpoints. Decentralized scheduling enables platforms to provide services in more regions, attracting more users and increasing overall revenue. Furthermore, decentralized scheduling enhances the platform’s flexibility in responding to demand fluctuations in different regions and time periods. When demand suddenly increases in a specific area, the platform can quickly reallocate resources to respond, improving service levels and customer satisfaction, thus generating more revenue overall. Second, when the benefits obtained from scheduling delivery boxes are high, centralized scheduling can achieve economies of scale. Centralized scheduling optimizes delivery routes and schedules, reducing logistics costs and improving delivery efficiency. For example, by using centralized distribution centers and fixed delivery routes, platforms can maximize resource utilization, lowering the cost per delivery [9]. When benefits are high, platforms can invest more resources in centralized scheduling. By consolidating resources, platforms can achieve more efficient management and operations. For instance, centralized scheduling reduces the complexity of inventory management, increases the utilization rate of delivery boxes, and reduces losses and waste. Centralized scheduling also improves service quality and enhances customer experience. Through centralized scheduling, platforms can better control delivery times and service quality, providing more consistent and efficient services, thereby increasing customer satisfaction and loyalty, and ultimately generating more revenue. Centralized scheduling allows better utilization of technology and data analysis. For example, platforms can use advanced scheduling algorithms and data analysis tools to optimize delivery routes and resource allocation, enhancing overall operational efficiency. The returns on technological investments are also greater in a centralized scheduling model, as centralized management can better leverage technological advantages. In conclusion, when the benefits obtained from scheduling delivery boxes are low, decentralized scheduling can maximize benefits for delivery platforms through risk distribution, market coverage, and flexible demand response; whereas, when the benefits are high, centralized scheduling can maximize benefits through economies of scale, resource integration, service quality improvement, and utilization of technology and data.

The following is an explanation of Conclusion 5. When the cost of scheduling delivery boxes is low and the returns are also low, catering enterprises can adopt demand-driven scheduling strategies to flexibly respond to market demand changes. Demand-driven scheduling can adjust delivery resources and strategies in real-time based on actual orders, meeting customer needs at minimal cost and maximizing overall returns. This approach better optimizes resource allocation. Since costs are low, catering enterprises can flexibly allocate boxes and delivery personnel according to demand fluctuations, avoiding resource wastage and over-allocation, thereby improving resource utilization and operational efficiency. In situations of low returns, catering enterprises can cover a broader market area through demand-driven scheduling, attracting more potential customers, increasing order volumes, and expanding market share. The flexibility of demand-driven scheduling allows enterprises to provide services in different regions and time periods, expanding their customer base [32]. When the cost of scheduling delivery boxes is low but the returns are high, catering enterprises can adopt centralized scheduling strategies to achieve higher operational efficiency through economies of scale. Centralized scheduling can optimize delivery routes and schedules, reducing delivery costs while improving delivery speed and service quality. This approach integrates internal and external resources, enhancing resource utilization [5]. Enterprises can establish centralized distribution centers to manage and dispatch boxes and delivery vehicles uniformly, maximizing resource utilization and controlling costs. Centralized scheduling can deliver higher service quality and consistency. In situations of high returns, catering enterprises can better control the delivery process through centralized scheduling, ensuring timely delivery of boxes and high-quality service, thereby enhancing customer satisfaction and loyalty. Centralized scheduling can better leverage advanced technologies and data analysis tools. Catering enterprises can use data analysis to predict demand, optimize scheduling strategies, and improve overall operational efficiency and returns. In a centralized scheduling model, the returns on technological investments are also more significant. In summary, when the cost of scheduling delivery boxes is low, demand-driven scheduling can maximize returns for catering enterprises through flexibility, resource optimization, and market coverage when returns are low. Conversely, when returns are high, centralized scheduling can maximize returns through economies of scale, resource integration, service quality improvement, and technology utilization.

The explanation of Conclusion 6 can be addressed from the following perspectives. When the cost of scheduling delivery boxes is high and the returns are low, decentralized scheduling can help catering enterprises spread risk. By scheduling in different regions and time periods, enterprises can avoid concentrating all resources in a specific area or time point, thereby reducing the risks associated with market fluctuations or demand uncertainties. With low returns, decentralized scheduling allows catering enterprises to cover more market areas, increasing potential customer reach. Although the returns from a single area might be low, covering multiple areas can increase overall returns, compensating for the low returns from individual areas. Decentralized scheduling enhances the flexibility of catering enterprises in responding to demand fluctuations in different regions and time periods. Enterprises can flexibly adjust delivery resources and strategies based on actual demand, ensuring timely and effective service in various regions, thus improving customer satisfaction and order volume. When the cost of scheduling delivery boxes is high and the returns are also high, centralized scheduling can achieve economies of scale, thereby reducing the unit cost. Through centralized distribution centers and optimized delivery routes, catering enterprises can reduce delivery time and resource waste, improve operational efficiency, and lower overall costs. Centralized scheduling integrates internal and external resources, maximizing resource utilization. Enterprises can manage and schedule boxes and delivery vehicles uniformly, reducing resource idleness and redundancy, thus achieving efficient resource utilization [23]. Centralized scheduling better controls the delivery process, ensuring service quality consistency. In high-return scenarios, enterprises can provide more efficient and stable delivery services through centralized scheduling, enhancing customer satisfaction and loyalty, thereby generating more orders and revenue. Centralized scheduling leverages advanced technologies and data analysis tools more effectively. Catering enterprises can predict demand through data analysis, optimize scheduling strategies, and improve overall operational efficiency [12]. In a centralized scheduling model, the returns on technological investments are also more significant, as centralized management can fully exploit technological advantages. In summary, when the cost of scheduling delivery boxes is high, decentralized scheduling can maximize returns for catering enterprises through risk distribution, market coverage, and flexible demand response when returns are low; whereas, when returns are high, centralized scheduling can maximize returns through economies of scale, resource integration, service quality improvement, and technology utilization. These two scheduling models have distinct advantages under different levels of returns and costs, catering to different operational needs and market environments.

## 5 . Conclusion

The efficient scheduling of shared takeaway food containers plays a crucial role in the sharing economy system. An efficient scheduling system can maximize the reuse of containers, reducing resource waste and environmental pollution. To study the applicability of different scheduling models for shared takeaway food containers, this paper constructs differential game models for centralized scheduling, decentralized scheduling, and demand-based scheduling. The equilibrium results are compared and analyzed. The research findings indicate that when the revenue from scheduling takeaway containers is low, decentralized scheduling can provide the maximum benefit for takeaway platforms; when the revenue is high, centralized scheduling offers the greatest benefit for takeaway platforms. For restaurants, when the revenue from scheduling is low and the costs incurred are minimal, demand-based scheduling can provide the maximum benefit; if the costs are high, decentralized scheduling offers the maximum benefit; otherwise, centralized scheduling can provide the greatest benefit.

This study can also be extended in several ways. For instance, the paper assumes that shared takeaway food containers are sterilized and disinfected, and there are no contamination issues during the scheduling process; a certain proportion of shared takeaway food containers are used locally; and information technology is fully applied in the area. In future research, these assumptions can be removed for further investigation. Additionally, some gaps in the current study can be addressed in future research. First, it is essential to determine the specific criteria adopted by takeaway platforms and restaurants in the scheduling of shared takeaway food containers under different conditions. Second, the outcomes of scheduling shared takeaway food containers can be translated into feasible policy recommendations for reference by takeaway platforms and restaurants. Third, during the scheduling of shared takeaway food containers in different regions, takeaway platforms and restaurants should establish a sequence of actions for relevant research, rather than taking actions simultaneously.

## Supporting information

S1 File Supporting information 1. (DOCX)

S2 File Supporting information 2. (DOCX)

S3 File Supporting information 3. (DOCX)
