# Supplementary material for: Centralized scheduling, decentralized scheduling or demand scheduling? How to more effectively allocate and recycle shared takeout lunch boxes
Source: PLoS One. 2025 Mar 4;20(3):e0319257. doi: 10.1371/journal.pone.0319257 (PMC11878947; doi:10.1371/journal.pone.0319257)
Supplement: S1 File — (DOCX) [file pone.0319257.s001.docx]

**Supporting information 1**

Take the derivatives of *F_C_*_1_ with respect to (13), and take the derivatives of *F_C_*_2_ with respect to (14), and set them equal to zero, we can get:

$F_{C1}^{*}\left(t\right)=\frac{a_{1}+a_{C}}{c_{1}}+\frac{f_{1}-f_{C}}{c_{1}}\frac{\partial V_{C1}}{\partial x_{C1}}$ (43)

$F_{C2}^{*}\left(t\right)=\left(\frac{a_{2}}{c_{2}}+\frac{f_{2}-f_{C}}{c_{2}}\frac{\partial V_{C2}}{\partial x_{C2}}\right)\ln\left(\text{e}+b_{C}\right)$ (44)

Substituting (43) into (13) and substituting (44) into (14), we can get:

$\begin{matrix} \rho V_{C1}=\left(a_{1}+a_{C}\right)\left(\frac{a_{1}+a_{C}}{c_{1}}+\frac{f_{1}-f_{C}}{c_{1}}\frac{\partial V_{C1}}{\partial x_{C1}}\right)-\frac{c_{1}}{2}\left(\frac{a_{1}+a_{C}}{c_{1}}+\frac{f_{1}-f_{C}}{c_{1}}\frac{\partial V_{C1}}{\partial x_{C1}}\right)^{2} \\ +lx_{C1}\left(t\right)+\frac{\partial V_{C1}}{\partial x_{C1}}\left[\left(f_{1}-f_{C}\right)\left(\frac{a_{1}+a_{C}}{c_{1}}+\frac{f_{1}-f_{C}}{c_{1}}\frac{\partial V_{C1}}{\partial x_{C1}}\right)-\delta x_{C1}\left(t\right)\right] \end{matrix}$ (45)

$\begin{matrix} \rho V_{C2}=a_{2}\left(\frac{a_{2}}{c_{2}}+\frac{f_{2}-f_{C}}{c_{2}}\frac{\partial V_{C2}}{\partial x_{C2}}\right)\ln\left(\text{e}+b_{C}\right)-\frac{c_{2}}{2\ln\left(\text{e}+b_{C}\right)}\left(\frac{a_{2}}{c_{2}}+\frac{f_{2}-f_{C}}{c_{2}}\frac{\partial V_{C2}}{\partial x_{C2}}\right)^{2}\left[\ln\left(\text{e}+b_{C}\right)\right]^{2} \\ +lx_{C2}\left(t\right)+\frac{\partial V_{C2}}{\partial x_{C2}}\left[\left(f_{2}-f_{C}\right)\left(\frac{a_{2}}{c_{2}}+\frac{f_{2}-f_{C}}{c_{2}}\frac{\partial V_{C2}}{\partial x_{C2}}\right)\ln\left(\text{e}+b_{C}\right)-\delta x_{C2}\left(t\right)\right] \end{matrix}$ (46)

Let $V_{C1}^{*}=k_{1}x_{C1}+k_{2}$, $V_{C2}^{\text{*}}=k_{3}x_{C2}+k_{4}$, wherein, *k*_1_, *k*_2_, *k*_3_ and *k*_4_ are all constants. The parameters of the optimal social welfare function can be obtained by calculation as follows:

$\left\{\begin{matrix} k_{1}=\frac{l}{\rho+\delta} \\ k_{2}=\frac{1}{\rho}\left(a_{1}+a_{C}\right)\left(\frac{a_{1}+a_{C}}{c_{1}}+\frac{f_{1}-f_{C}}{c_{1}}\frac{l}{\rho+\delta}\right)-\frac{c_{1}}{2}\frac{1}{\rho}\left(\frac{a_{1}+a_{C}}{c_{1}}+\frac{f_{1}-f_{C}}{c_{1}}\frac{l}{\rho+\delta}\right)^{2} \\ +\frac{l}{\rho+\delta}\frac{1}{\rho}\left(f_{1}-f_{C}\right)\left(\frac{a_{1}+a_{C}}{c_{1}}+\frac{f_{1}-f_{C}}{c_{1}}\frac{l}{\rho+\delta}\right) \end{matrix}\right.$ (47)

$\left\{\begin{matrix} k_{3}=\frac{l}{\rho+\delta} \\ k_{4}=\frac{1}{\rho}a_{2}\left(\frac{a_{2}}{c_{2}}+\frac{f_{2}-f_{C}}{c_{2}}\frac{l}{\rho+\delta}\right)\ln\left(\text{e}+b_{C}\right)-\frac{1}{\rho}\frac{c_{2}}{2\ln\left(\text{e}+b_{C}\right)}\left(\frac{a_{2}}{c_{2}}+\frac{f_{2}-f_{C}}{c_{2}}\frac{l}{\rho+\delta}\right)^{2}\left[\ln\left(\text{e}+b_{C}\right)\right]^{2} \\ +\frac{1}{\rho}\frac{l}{\rho+\delta}\left(f_{2}-f_{C}\right)\left(\frac{a_{2}}{c_{2}}+\frac{f_{2}-f_{C}}{c_{2}}\frac{l}{\rho+\delta}\right)\ln\left(\text{e}+b_{C}\right) \end{matrix}\right.$(48)

Therefore, it can be concluded that:

$\begin{matrix} V_{C1}^{*}=\frac{l}{\rho+\delta}x_{C1}+\frac{1}{\rho}\left(a_{1}+a_{C}\right)\left(\frac{a_{1}+a_{C}}{c_{1}}+\frac{f_{1}-f_{C}}{c_{1}}\frac{l}{\rho+\delta}\right)-\frac{c_{1}}{2}\frac{1}{\rho}\left(\frac{a_{1}+a_{C}}{c_{1}}+\frac{f_{1}-f_{C}}{c_{1}}\frac{l}{\rho+\delta}\right)^{2} \\ +\frac{l}{\rho+\delta}\frac{1}{\rho}\left(f_{1}-f_{C}\right)\left(\frac{a_{1}+a_{C}}{c_{1}}+\frac{f_{1}-f_{C}}{c_{1}}\frac{l}{\rho+\delta}\right) \end{matrix}$ (49)

$\begin{matrix} V_{C2}^{\text{*}}=\frac{1}{\rho}a_{2}\left(\frac{a_{2}}{c_{2}}+\frac{f_{2}-f_{C}}{c_{2}}\frac{l}{\rho+\delta}\right)\ln\left(\text{e}+b_{C}\right)-\frac{1}{\rho}\frac{c_{2}}{2\ln\left(\text{e}+b_{C}\right)}\left(\frac{a_{2}}{c_{2}}+\frac{f_{2}-f_{C}}{c_{2}}\frac{l}{\rho+\delta}\right)^{2}\left[\ln\left(\text{e}+b_{C}\right)\right]^{2} \\ +\frac{1}{\rho}\frac{l}{\rho+\delta}\left(f_{2}-f_{C}\right)\left(\frac{a_{2}}{c_{2}}+\frac{f_{2}-f_{C}}{c_{2}}\frac{l}{\rho+\delta}\right)\ln\left(\text{e}+b_{C}\right)+\frac{l}{\rho+\delta}x_{C2} \end{matrix}$(50)

In this case,

$F_{C1}^{*}\left(t\right)=\frac{a_{1}+a_{C}}{c_{1}}+\frac{f_{1}-f_{C}}{c_{1}}\frac{l}{\rho+\delta}$ (51)

$F_{C2}^{*}\left(t\right)=\left(\frac{a_{2}}{c_{2}}+\frac{f_{2}-f_{C}}{c_{2}}\frac{l}{\rho+\delta}\right)\ln\left(\text{e}+b_{C}\right)$ (52)
