# Supplementary material for: Centralized scheduling, decentralized scheduling or demand scheduling? How to more effectively allocate and recycle shared takeout lunch boxes
Source: PLoS One. 2025 Mar 4;20(3):e0319257. doi: 10.1371/journal.pone.0319257 (PMC11878947; doi:10.1371/journal.pone.0319257)
Supplement: S2 File — (DOCX) [file pone.0319257.s002.docx]

**Supporting information 2**

Take the derivatives of *F_D_*_1_ with respect to (15), and take the derivatives of *F_D_*_2_ with respect to (16), and set them equal to zero, we can get:

$F_{D1}^{*}\left(t\right)=\frac{a_{1}-a_{D}}{c_{1}}+\frac{f_{1}+f_{D}}{c_{1}}\frac{\partial V_{D1}}{\partial x_{D1}}$ (53)

$F_{D2}^{\text{*}}\left(t\right)=\frac{a_{2}}{c_{2}+c_{D}}+\frac{f_{2}+f_{D}}{c_{2}+c_{D}}\frac{\partial V_{D2}}{\partial x_{D2}}$ (54)

Substituting (53) into (15) and substituting (54) into (16), we can get:

$\begin{matrix} \rho V_{D1}=\left(a_{1}-a_{D}\right)\left(\frac{a_{1}-a_{D}}{c_{1}}+\frac{f_{1}+f_{D}}{c_{1}}\frac{\partial V_{D1}}{\partial x_{D1}}\right)-\frac{c_{1}}{2}\left(\frac{a_{1}-a_{D}}{c_{1}}+\frac{f_{1}+f_{D}}{c_{1}}\frac{\partial V_{D1}}{\partial x_{D1}}\right)^{2}+lx_{D1}\left(t\right) \\ +\frac{\partial V_{D1}}{\partial x_{D1}}\left[\left(f_{1}+f_{D}\right)\left(\frac{a_{1}-a_{D}}{c_{1}}+\frac{f_{1}+f_{D}}{c_{1}}\frac{\partial V_{D1}}{\partial x_{D1}}\right)-\delta x_{D1}\left(t\right)\right] \end{matrix}$ (55)

$\begin{matrix} \rho V_{D2}=a_{2}\left(\frac{a_{2}}{c_{2}+c_{D}}+\frac{f_{2}+f_{D}}{c_{2}+c_{D}}\frac{\partial V_{D2}}{\partial x_{D2}}\right)-\frac{\left(c_{2}+c_{D}\right)}{2}\left(\frac{a_{2}}{c_{2}+c_{D}}+\frac{f_{2}+f_{D}}{c_{2}+c_{D}}\frac{\partial V_{D2}}{\partial x_{D2}}\right)^{2}+lx_{D2}\left(t\right) \\ +\frac{\partial V_{D2}}{\partial x_{D2}}\left[\left(f_{2}+f_{D}\right)\left(\frac{a_{2}}{c_{2}+c_{D}}+\frac{f_{2}+f_{D}}{c_{2}+c_{D}}\frac{\partial V_{D2}}{\partial x_{D2}}\right)-\delta x_{D2}\left(t\right)\right] \end{matrix}$ (56)

Let $V_{D1}^{*}=k_{5}x_{D1}+k_{6}$,$V_{D2}^{\text{*}}=k_{7}x_{D2}+k_{8}$, wherein, *k*_5_, *k*_6_, *k*_7_ and *k*_8_ are all constants. The parameters of the optimal social welfare function can be obtained by calculation as follows:

$\left\{\begin{matrix} k_{5}=\frac{l}{\rho+\delta} \\ k_{6}=\frac{1}{\rho}\left(a_{1}-a_{D}\right)\left(\frac{a_{1}-a_{D}}{c_{1}}+\frac{f_{1}+f_{D}}{c_{1}}\frac{l}{\rho+\delta}\right)-\frac{c_{1}}{2}\frac{1}{\rho}\left(\frac{a_{1}-a_{D}}{c_{1}}+\frac{f_{1}+f_{D}}{c_{1}}\frac{l}{\rho+\delta}\right)^{2} \\ +\frac{1}{\rho}\frac{l}{\rho+\delta}\left(f_{1}+f_{D}\right)\left(\frac{a_{1}-a_{D}}{c_{1}}+\frac{f_{1}+f_{D}}{c_{1}}\frac{l}{\rho+\delta}\right) \end{matrix}\right.$ (57)

$\left\{\begin{matrix} k_{7}=\frac{l}{\rho+\delta} \\ k_{8}=\frac{1}{\rho}a_{2}\left(\frac{a_{2}}{c_{2}+c_{D}}+\frac{f_{2}+f_{D}}{c_{2}+c_{D}}\frac{l}{\rho+\delta}\right)-\frac{\left(c_{2}+c_{D}\right)}{2}\frac{1}{\rho}\left(\frac{a_{2}}{c_{2}+c_{D}}+\frac{f_{2}+f_{D}}{c_{2}+c_{D}}\frac{l}{\rho+\delta}\right)^{2} \\ +\frac{l}{\rho+\delta}\frac{1}{\rho}\left(f_{2}+f_{D}\right)\left(\frac{a_{2}}{c_{2}+c_{D}}+\frac{f_{2}+f_{D}}{c_{2}+c_{D}}\frac{l}{\rho+\delta}\right) \end{matrix}\right.$ (58)

Therefore, it can be concluded that:

$\begin{matrix} V_{D1}^{*}=\frac{l}{\rho+\delta}x_{D1}+\frac{1}{\rho}\left(a_{1}-a_{D}\right)\left(\frac{a_{1}-a_{D}}{c_{1}}+\frac{f_{1}+f_{D}}{c_{1}}\frac{l}{\rho+\delta}\right)-\frac{c_{1}}{2}\frac{1}{\rho}\left(\frac{a_{1}-a_{D}}{c_{1}}+\frac{f_{1}+f_{D}}{c_{1}}\frac{l}{\rho+\delta}\right)^{2} \\ +\frac{1}{\rho}\frac{l}{\rho+\delta}\left(f_{1}+f_{D}\right)\left(\frac{a_{1}-a_{D}}{c_{1}}+\frac{f_{1}+f_{D}}{c_{1}}\frac{l}{\rho+\delta}\right) \end{matrix}$ (59)

$\begin{matrix} V_{D2}^{\text{*}}=\frac{l}{\rho+\delta}x_{D2}+\frac{1}{\rho}a_{2}\left(\frac{a_{2}}{c_{2}+c_{D}}+\frac{f_{2}+f_{D}}{c_{2}+c_{D}}\frac{l}{\rho+\delta}\right)-\frac{\left(c_{2}+c_{D}\right)}{2}\frac{1}{\rho}\left(\frac{a_{2}}{c_{2}+c_{D}}+\frac{f_{2}+f_{D}}{c_{2}+c_{D}}\frac{l}{\rho+\delta}\right)^{2} \\ +\frac{l}{\rho+\delta}\frac{1}{\rho}\left(f_{2}+f_{D}\right)\left(\frac{a_{2}}{c_{2}+c_{D}}+\frac{f_{2}+f_{D}}{c_{2}+c_{D}}\frac{l}{\rho+\delta}\right) \end{matrix}$ (60)

In this case,

$F_{D1}^{*}\left(t\right)=\frac{a_{1}-a_{D}}{c_{1}}+\frac{f_{1}+f_{D}}{c_{1}}\frac{l}{\rho+\delta}$ (61)

$F_{D2}^{\text{*}}\left(t\right)=\frac{a_{2}}{c_{2}+c_{D}}+\frac{f_{2}+f_{D}}{c_{2}+c_{D}}\frac{l}{\rho+\delta}$ (62)
