# Supplementary material for: Centralized scheduling, decentralized scheduling or demand scheduling? How to more effectively allocate and recycle shared takeout lunch boxes
Source: PLoS One. 2025 Mar 4;20(3):e0319257. doi: 10.1371/journal.pone.0319257 (PMC11878947; doi:10.1371/journal.pone.0319257)
Supplement: S3 File — (DOCX) [file pone.0319257.s003.docx]

**Supporting information 3**

Take the derivatives of *F_B_*_1_ with respect to (17), and take the derivatives of *F_B_*_2_ with respect to (18), and set them equal to zero, we can get:

$F_{B1}^{\text{*}}\left(t\right)\text{=}\frac{a_{1}+a_{B}}{c_{1}+c_{B}}+\frac{f_{1}}{c_{1}+c_{B}}\frac{\partial V_{B1}}{\partial x_{B1}}$ (63)

$F_{B2}^{\text{*}}\left(t\right)\text{=}\frac{a_{2}}{c_{2}}+\frac{f_{2}}{c_{2}}\frac{\partial V_{B2}}{\partial x_{B2}}$ (64)

Substituting (63) into (17) and substituting (64) into (18), we can get:

$\begin{matrix} \rho V_{B1}=\left(a_{1}+a_{B}\right)\left(\frac{a_{1}+a_{B}}{c_{1}+c_{B}}+\frac{f_{1}}{c_{1}+c_{B}}\frac{\partial V_{B1}}{\partial x_{B1}}\right)-\frac{c_{1}+c_{B}}{2}\left(\frac{a_{1}+a_{B}}{c_{1}+c_{B}}+\frac{f_{1}}{c_{1}+c_{B}}\frac{\partial V_{B1}}{\partial x_{B1}}\right)^{2}+lx_{B1}\left(t\right) \\ +\frac{\partial V_{B1}}{\partial x_{B1}}\left[f_{1}\left(\frac{a_{1}+a_{B}}{c_{1}+c_{B}}+\frac{f_{1}}{c_{1}+c_{B}}\frac{\partial V_{B1}}{\partial x_{B1}}\right)-\delta x_{B1}\left(t\right)\right] \end{matrix}$ (65)

$\rho V_{B2}=a_{2}\left(\frac{a_{2}}{c_{2}}+\frac{f_{2}}{c_{2}}\frac{\partial V_{B2}}{\partial x_{B2}}\right)-\frac{c_{2}}{2}\left(\frac{a_{2}}{c_{2}}+\frac{f_{2}}{c_{2}}\frac{\partial V_{B2}}{\partial x_{B2}}\right)^{2}+lx_{F2}\left(t\right)+\frac{\partial V_{B2}}{\partial x_{B2}}\left[f_{2}\left(\frac{a_{2}}{c_{2}}+\frac{f_{2}}{c_{2}}\frac{\partial V_{B2}}{\partial x_{B2}}\right)-\delta x_{B2}\left(t\right)\right]$ (66)

Let $V_{B1}^{*}=k_{9}x_{B1}+k_{10}$,$V_{B2}^{\text{*}}=k_{11}x_{B2}+k_{12}$, wherein, *k*_9_, *k*_10_, *k*_11_ and *k*_12_ are all constants. The parameters of the optimal social welfare function can be obtained by calculation as follows:

$\left\{\begin{matrix} k_{9}=\frac{l}{\rho+\delta} \\ k_{10}=\frac{1}{\rho}\left(a_{1}+a_{B}\right)\left(\frac{a_{1}+a_{B}}{c_{1}+c_{B}}+\frac{f_{1}}{c_{1}+c_{B}}\frac{l}{\rho+\delta}\right)-\frac{c_{1}+c_{B}}{2}\frac{1}{\rho}\left(\frac{a_{1}+a_{B}}{c_{1}+c_{B}}+\frac{f_{1}}{c_{1}+c_{B}}\frac{l}{\rho+\delta}\right)^{2} \\ +\frac{l}{\rho+\delta}\frac{1}{\rho}f_{1}\left(\frac{a_{1}+a_{B}}{c_{1}+c_{B}}+\frac{f_{1}}{c_{1}+c_{B}}\frac{l}{\rho+\delta}\right) \end{matrix}\right.$ (67)

$\left\{\begin{matrix} k_{11}=\frac{l}{\rho+\delta} \\ k_{12}=\frac{1}{\rho}a_{2}\left(\frac{a_{2}}{c_{2}}+\frac{f_{2}}{c_{2}}\frac{l}{\rho+\delta}\right)-\frac{c_{2}}{2}\frac{1}{\rho}\left(\frac{a_{2}}{c_{2}}+\frac{f_{2}}{c_{2}}\frac{l}{\rho+\delta}\right)^{2}+\frac{l}{\rho+\delta}\frac{1}{\rho}f_{2}\left(\frac{a_{2}}{c_{2}}+\frac{f_{2}}{c_{2}}\frac{l}{\rho+\delta}\right) \end{matrix}\right.$ (68)

Therefore, it can be concluded that:

$\begin{matrix} V_{B1}^{*}=\frac{l}{\rho+\delta}x_{B1}+\frac{1}{\rho}\left(a_{1}+a_{B}\right)\left(\frac{a_{1}+a_{B}}{c_{1}+c_{B}}+\frac{f_{1}}{c_{1}+c_{B}}\frac{l}{\rho+\delta}\right)-\frac{c_{1}+c_{B}}{2}\frac{1}{\rho}\left(\frac{a_{1}+a_{B}}{c_{1}+c_{B}}+\frac{f_{1}}{c_{1}+c_{B}}\frac{l}{\rho+\delta}\right)^{2} \\ +\frac{l}{\rho+\delta}\frac{1}{\rho}f_{1}\left(\frac{a_{1}+a_{B}}{c_{1}+c_{B}}+\frac{f_{1}}{c_{1}+c_{B}}\frac{l}{\rho+\delta}\right) \end{matrix}$ (69)

$V_{B2}^{\text{*}}=\frac{l}{\rho+\delta}x_{B2}+\frac{1}{\rho}a_{2}\left(\frac{a_{2}}{c_{2}}+\frac{f_{2}}{c_{2}}\frac{l}{\rho+\delta}\right)-\frac{c_{2}}{2}\frac{1}{\rho}\left(\frac{a_{2}}{c_{2}}+\frac{f_{2}}{c_{2}}\frac{l}{\rho+\delta}\right)^{2}+\frac{l}{\rho+\delta}\frac{1}{\rho}f_{2}\left(\frac{a_{2}}{c_{2}}+\frac{f_{2}}{c_{2}}\frac{l}{\rho+\delta}\right)$ (70)

In this case,

$F_{B1}^{\text{*}}\left(t\right)\text{=}\frac{a_{1}+a_{B}}{c_{1}+c_{B}}+\frac{f_{1}}{c_{1}+c_{B}}\frac{l}{\rho+\delta}$ (71)

$F_{B2}^{\text{*}}\left(t\right)\text{=}\frac{a_{2}}{c_{2}}+\frac{f_{2}}{c_{2}}\frac{l}{\rho+\delta}$ (72)
